# Support surfaces for pressure ulcer prevention: A network meta-analysis

**DOI:** 10.1371/journal.pone.0192707

**Published:** 2018-02-23

**Authors:** Chunhu Shi, Jo C. Dumville, Nicky Cullum

**Affiliations:** 1 Division of Nursing, Midwifery & Social Work, School of Health Sciences, Faculty of Biology, Medicine & Health, University of Manchester, Manchester Academic Health Science Centre, Manchester, United Kingdom; 2 Research and Innovation Division, Manchester University NHS Foundation Trust, Manchester Academic Health Science Centre, Manchester, United Kingdom; University of Illinois at Urbana-Champaign, UNITED STATES

## Abstract

**Background:**

Pressure ulcers are a prevalent and global issue and support surfaces are widely used for preventing ulceration. However, the diversity of available support surfaces and the lack of direct comparisons in RCTs make decision-making difficult.

**Objectives:**

To determine, using network meta-analysis, the relative effects of different support surfaces in reducing pressure ulcer incidence and comfort and to rank these support surfaces in order of their effectiveness.

**Methods:**

We conducted a systematic review, using a literature search up to November 2016, to identify randomised trials comparing support surfaces for pressure ulcer prevention. Two reviewers independently performed study selection, risk of bias assessment and data extraction. We grouped the support surfaces according to their characteristics and formed evidence networks using these groups. We used network meta-analysis to estimate the relative effects and effectiveness ranking of the groups for the outcomes of pressure ulcer incidence and participant comfort. GRADE was used to assess the certainty of evidence.

**Main results:**

We included 65 studies in the review. The network for assessing pressure ulcer incidence comprised evidence of low or very low certainty for most network contrasts. There was moderate-certainty evidence that powered active air surfaces and powered hybrid air surfaces probably reduce pressure ulcer incidence compared with standard hospital surfaces (risk ratios (RR) 0.42, 95% confidence intervals (CI) 0.29 to 0.63; 0.22, 0.07 to 0.66, respectively). The network for comfort suggested that powered active air-surfaces are probably slightly less comfortable than standard hospital mattresses (RR 0.80, 95% CI 0.69 to 0.94; moderate-certainty evidence).

**Conclusions:**

This is the first network meta-analysis of the effects of support surfaces for pressure ulcer prevention. Powered active air-surfaces probably reduce pressure ulcer incidence, but are probably less comfortable than standard hospital surfaces. Most prevention evidence was of low or very low certainty, and more research is required to reduce these uncertainties.

## Introduction

Pressure ulcers are localised injuries to the skin and/or underlying tissue, which are also known as pressure injuries, pressure sores, decubitus ulcers and bedsores [1]. Pressure ulcers represent a serious heath burden with a point prevalence of approximately 3.1 per 10,000 in the United Kingdom (UK) [2]. It has been estimated that the treatment of pressure ulcers costs approximately 4% (between £1.4 and £2.1 billion) of the total health budget of the UK (1999/2000 financial year) [3].

Pressure ulcers are caused by localised pressure and shear [1], thus intervention to alleviate pressure and shear is an important part of pressure ulcer prevention. Support surfaces (e.g. mattresses, overlays, integrated bed systems) are designed to work towards preventing pressure ulcers primarily in this way [4]. Various types of support surfaces have been developed with different mechanisms for pressure and shear relief including (1) redistributing the weight over the maximum body surface area; (2) mechanically alternating the pressure beneath body to reduce the duration of the applied pressure [5]; or (3) redistributing pressure by a combination of the above, allowing health care professionals to change the mode according to a person’s needs [6]. Support surfaces are made from a variety of construction materials (e.g. foam) and have different functional features (e.g. low-air-loss) [4]. Identification of the optimum support surface from the diverse options available requires evidence on their relative effectiveness in terms of how well they prevent the incidence of new pressure ulcers [2].

Currently, seven systematic reviews containing meta-analyses have summarised randomised controlled trial (RCT) and quasi-randomised trial evidence to inform choice of support surface [7–13]. Of these reviews, one high-quality Cochrane review includes all studies covered by the remaining six reviews and offers the most comprehensive summary of current evidence [9]. However, all these reviews (including the Cochrane review [9]) use an outdated support surface classification systems [5] now superseded by the recent internationally agreed NPUAP Support Surface Standards Initiative (S3I) classification system [4]. Additionally, the reviews all use pairwise meta-analysis to synthesise evidence for head-to-head comparisons of support surfaces. There remains a lack of evidence on the relative effects of different support surfaces, in part due to a lack of head-to-head RCT data across the plethora of treatment options available.

To tackle this problem, an advanced meta-analysis technique, network meta-analysis, can be employed. The approach can simultaneously compare multiple competing interventions in a single statistical model whilst maintaining randomisation as with standard meta-analysis [14–16]. The network meta-analysis has the following advantages. Firstly network meta-analysis can produce “indirect evidence” for a potential comparison where a head-to-head comparison is unavailable. A network can be developed to link the direct evidence of, say, A vs. B and B vs. C (i.e. evidence from studies with A vs. B and B vs. C as head-to-head comparisons), via a common comparator (i.e. B in this example) to derive an indirect estimate of A vs. C. Secondly, both indirect and direct evidence can be used together which then improves the precision of effect estimates. Thirdly, effect estimates from network meta-analysis can be linked to probabilistic modelling to allow the ranking of treatments based on which is likely to be the most effective for the outcome of interest, which is likely to be the second best and so on. This is a valuable approach for considering the results of the network across multiple interventions in a single measure [14–16].

The aim of this work was to synthesise the available evidence from RCTs in a network meta-analysis to: (1) assess the relative effects of different classes of support surfaces for reducing pressure ulcer incidence in adults in any setting; (2) to assess the relative effects of different classes of support surface in terms of reported comfort; and (3) to rank all classes of support surface in order of effectiveness regarding pressure ulcer prevention.

## Methods

This review was preceded by a protocol and registered prospectively in PROSPERO (CRD42016042154). This report complies with the relevant PRISMA extension statement [17] (see S1 File).

### Search strategy

As the most comprehensive summary of available evidence in the topic of our review, the current Cochrane review had identified and included 59 RCTs and quasi-randomised trials comparing support surfaces for pressure ulcer prevention, with a database search up to April 2015 [9].

We performed an update search of the following databases for the current Cochrane review: the Cochrane Wounds Specialised Register (10 August 2016); the Cochrane Central Register of Controlled Trials (CENTRAL) (2016, Issue 7); Ovid MEDLINE (1946 to 10 August 2016); Ovid EMBASE (1974 to 10 August 2016); EBSCO CINAHL Plus (1937 to 10 August 2016). Additionally, we searched the Chinese Biomedical Literature Database (1978 to 30 November 2016). There was no restriction on the basis of language or publication status (see S2 File for Ovid MEDLINE Search Strategy).

We also searched other resources: ClinicalTrials.gov and WHO International Clinical Trials Registry Platform (ICTRP) (24 August 2016), the Journal of Tissue Viability via hand-searching (1991 to November 2016), and the reference lists of seven previously published systematic reviews [7–13].

### Eligibility criteria

We included published and unpublished RCTs, comparing pressure-redistribution support surfaces—mattresses, overlays, and integrated bed systems—in adults at risk of pressure ulcer development, in any setting. We excluded studies of seating and cushions, limb protectors, turning beds, traditional Chinese medicine-related surfaces and home-made support surfaces. Recent concern about the validity of RCTs from China led us to only consider those with full descriptions of robust randomisation methods (e.g. random number tables) as eligible [18, 19].

Our primary outcome was pressure ulcer incidence. We considered this outcome as either the proportion of participants developing a new ulcer at the latest trial follow-up point (or the pre-specified time point of primary focus if this was different to the longest follow-up point) or time-to-pressure ulcer incidence. The secondary outcome was patient-reported comfort on support surface (measured as the proportion of patients reporting comfort).

### Selection of studies

Two reviewers independently assessed the titles and abstracts of the search results for relevance and then independently inspected the full text of all potentially eligible studies. Because the non-Chinese database search was an updated search of the Cochrane review published by McInnes and colleagues [9], all studies included by the Cochrane review were checked again for relevance. Disagreements were resolved by discussion between the two reviewers and involvement of a third reviewer if necessary.

### Data extraction

Where eligible studies had been previously included in McInnes *et al* [9], one reviewer checked the original data extraction of these studies and extracted additional data where necessary, and another reviewer checked all data. Two reviewers independently extracted data for new included studies. Any disagreements were resolved by discussion and, if necessary, with the involvement of a third reviewer. Where necessary, the authors of included studies were contacted to collect and/or clarify data.

The following data were extracted using a pre-prepared data extraction form: basic characteristics of studies (e.g. country, setting, and funding sources); characteristics of participants (including eligibility criteria, average age, proportions of participants by gender, and participants’ baseline skin status); description of support surfaces and details on any co-interventions; number randomised, follow-up durations; drop-outs; primary and secondary outcome data.

In order to assign support surfaces to intervention groups, we extracted full descriptions from included studies where possible. However, when necessary we supplemented the information provided with that from external sources such as other publications about the same support surface, manufacturers’ and/or product websites and expert clinical opinion [20].

#### Classification of interventions

Support surfaces in included studies were classified using the NPUAP system [4] and assigned to one of 14 intervention groups [21] (see S3 File for the detailed steps and Table 1 for the 14 intervention groups).

**Table 1:** 14 intervention groups, explanations and selected examples from included studies.

Intervention groups	Reviewers’ explanations	Selected examples (with support surface brands if possible)
Powered/non-powered reactive air surfaces	A group of support surfaces constructed of air-cells, which redistribute body weight over a maximum surface area (i.e. has reactive pressure redistribution mode), with or without the requirement for electrical power	Static air mattress overlay, dry flotation mattress (e.g., Roho, Sofflex), static air mattress (e.g., EHOB), and static mode of Duo 2 mattress
Powered/non-powered reactive low-air-loss air surfaces	A group of support surfaces made of air-cells, which have reactive pressure redistribution modes and a low-air-loss function, with or without the requirement for electrical power	Low-air-loss Hydrotherapy
Powered reactive air-fluidised surfaces	A group of support surfaces made of air-cells, which have reactive pressure redistribution modes and an air-fluidised function, with the requirement for electrical power	Air-fluidised bed (e.g., Clinitron)
Non-powered reactive foam surfaces	A group of support surfaces made of foam materials, which have a reactive pressure redistribution function, without the requirement for electrical power	Convoluted foam overlay (or pad), elastic foam overlay (e.g., Aiartex, microfluid static overlay), polyether foam pad, foam mattress replacement (e.g. MAXIFLOAT), solid foam overlay, viscoelastic foam mattress/overlay (e.g., Tempur, CONFOR-Med, Akton, Thermo)
Non-powered reactive fibre surfaces	A group of support surfaces made of fibre materials, which have a reactive pressure redistribution function, without the requirement for electrical power	Silicore (e.g., Spenco) overlay/pad
Non-powered reactive gel surfaces	A group of support surfaces made of gel materials, which have a reactive pressure redistribution function, without the requirement for electrical power	Gel mattress, gel pad used in operating theatre
Non-powered reactive sheepskin surfaces	A group of support surfaces made of sheepskin, which have a reactive pressure redistribution function, without the requirement for electrical power	Australian Medical Sheepskins overlay
Non-powered reactive water surfaces	A group of support surfaces based on water, which has the capability of a reactive pressure redistribution function, without the requirement for electrical power	Water mattress
Powered active air surfaces	A group of support surfaces made of air-cells, which mechanically alternate the pressure beneath the body to reduce the duration of the applied pressure (mainly via inflating and deflating to alternately change the contact area between support surfaces and the body) (i.e. alternating pressure (or active) mode), with the requirement for electrical power	Alternating pressure-relieving air mattress (e.g., Nimbus II, Cairwave, Airwave, MicroPulse), large-celled ripple
Powered active air surfaces and non-powered reactive foam surfaces	A group of support surfaces which use powered active air surfaces and non-powered reactive foam surfaces in combination	Alternating pressure-relieving air mattress in combination with viscoelastic foam mattress/overlay (e.g., Nimbus plus Tempur)
Powered active low-air-loss air surfaces	A group of support surfaces made of air-cells, which have the capability of alternating pressure redistribution as well as low-air-loss for drying local skin, with the requirement for electrical power	Alternating pressure low-air-loss air mattress
Powered hybrid system air surfaces	A group of support surfaces made of air-cells, which offer both reactive and active pressure redistribution modes, with the requirement for electrical power	Foam mattress with dynamic and static modes (e.g. Softform Premier Active)
Powered hybrid system low-air-loss air surfaces	A group of support surfaces made of air-cells, which offer both reactive and active pressure redistribution modes as well as a low-air-loss function, with the requirement for electrical power	Stand-alone bed unit with alternating pressure, static modes and low air-loss (e.g., TheraPulse)
Standard hospital surfaces	A group of support surfaces made of any materials, used as usual in a hospital and without reactive nor active pressure redistribution capabilities, nor any other functions (e.g. low-air-loss, or air-fluidised).	Standard hospital (foam) mattress, NHS Contract hospital mattress, standard operating theatre surface configuration, standard bed unit and usual care

### Risk of bias assessment

We used Cochrane’s Risk of Bias tool to assess risk of bias of each included study [22]. For new included studies, two reviewers independently assessed **domain-specific risk of bias** [22]. For studies included by McInnes and colleagues [9], previous judgements were checked by two reviewers independently and, where required, updated. Any discrepancy between two reviewers was resolved by discussion and a third reviewer where necessary.

We then followed GRADE principles to summarise the **overall risk of bias** across domains for each included study [23]. After this, we applied the approach proposed by Salanti and colleagues [24] to judge the **overall risk of bias** (referred to hereon as “study limitations”) for direct evidence (i.e. pairwise meta-analysis), network contrasts, and the entire network. Three categories were used to qualitatively rate study limitations: no serious limitations; serious limitations; and very serious limitation.

### Data synthesis and analyses

We conducted all meta-analyses based on a frequentist framework with a random effects model [25]. All estimates are presented as risk ratios (RR) with 95% confidence intervals (CIs). When presenting summaries of findings, we also calculated the absolute risk of an event for a specific intervention group compared with that for a standard hospital surface. The baseline risk used was the outcome on standard hospital surfaces (the median risk across studies that provided data for the outcome).

We performed pairwise meta-analyses in RevMan, calculated I-squared (I^2^) measures and visually inspected the forest plots to assess statistical heterogeneity [26]. We then conducted network meta-analysis in STATA® (StataCorp. 2013) using published network commands and network graph packages [27, 28] (see S4 File for STATA commands used in the review). A consistency model was fitted to estimate relative effects [29]. Following this, we calculated the relative rankings of intervention groups and presented the surface under the cumulative ranking curve (SUCRA) percentages [27]. For any outcome, we performed network meta-analysis only if intervention groups could be connected to form a network; however, we did not exclude comparisons of support surfaces assigned to the same group from the overall systematic review. The full dataset is available on request.

We assessed the transitivity assumption for each network by comparing the similarities of study-level characteristics across direct comparisons within the network [30]. When data were insufficient for this assessment, we assumed that the transitivity assumption was met. Inconsistency between direct and indirect evidence was examined globally by running the design-by-treatment interaction model and locally by using the node-splitting method and inconsistency plot test [28, 31–33]. We also explored the sensitivity of the global inconsistency finding to alternative modelling approaches by running a post hoc sensitivity analysis using the model of Lu and Ades [34]. It is worth noting that because the model of Lu and Ades [34] depends on the ordering of treatments in the presence of multi-arm studies [28] the design-by-treatment interaction model was used in the main analysis. We then evaluated the common network heterogeneity using the tau-squared (tau^2^) and the I^2^ measure and the 95% CIs of I^2^, and decomposed the common network heterogeneity to inconsistency and within-study heterogeneity in R to locate the source of heterogeneity [35]. The heterogeneity was considered as low, moderate, or high if I^2^ = 25%, 50%, or 75%, respectively [36].

When important inconsistency and/or heterogeneity occurred, we followed steps proposed by Cipriani and colleagues [37] to investigate further. Of these steps, we performed pre-specified subgroup analyses for funding sources [38] and risk of bias [39]; as well as four exploratory sub-group analyses: setting, considering operating theatre as setting or not, baseline skin status, and follow-up duration. Additionally, we performed one sensitivity analysis to assess the impact of missing data (i.e. a complete case analysis for the main analysis, followed by a repeated analysis with missing data added to the denominator but not the numerator) and another one for the impact of unpublished studies by removing them from the analysis.

### Assessing the certainty of evidence

We assessed the potential for publication bias by considering the completeness of the literature search (i.e. inspecting the scope of the literature search, and assessing the volume of unpublished data located), and plotting the funnel plot for each pairwise meta-analysis that included more than 10 studies and a comparison-adjusted funnel plot for the network [24, 27, 40]. To obtain a meaningful comparison-adjusted funnel plot, we ordered the intervention groups by assuming that small studies are likely to favour advanced support surfaces [27]. Finally, we followed the GRADE approach proposed by Salanti and colleagues [24] to assess the certainty of evidence from the network meta-analysis for each network contrast and the ranking of intervention groups: the overall certainty could be rated from high, moderate, low to very low.

## Results

### Search results

**Fig 1:**
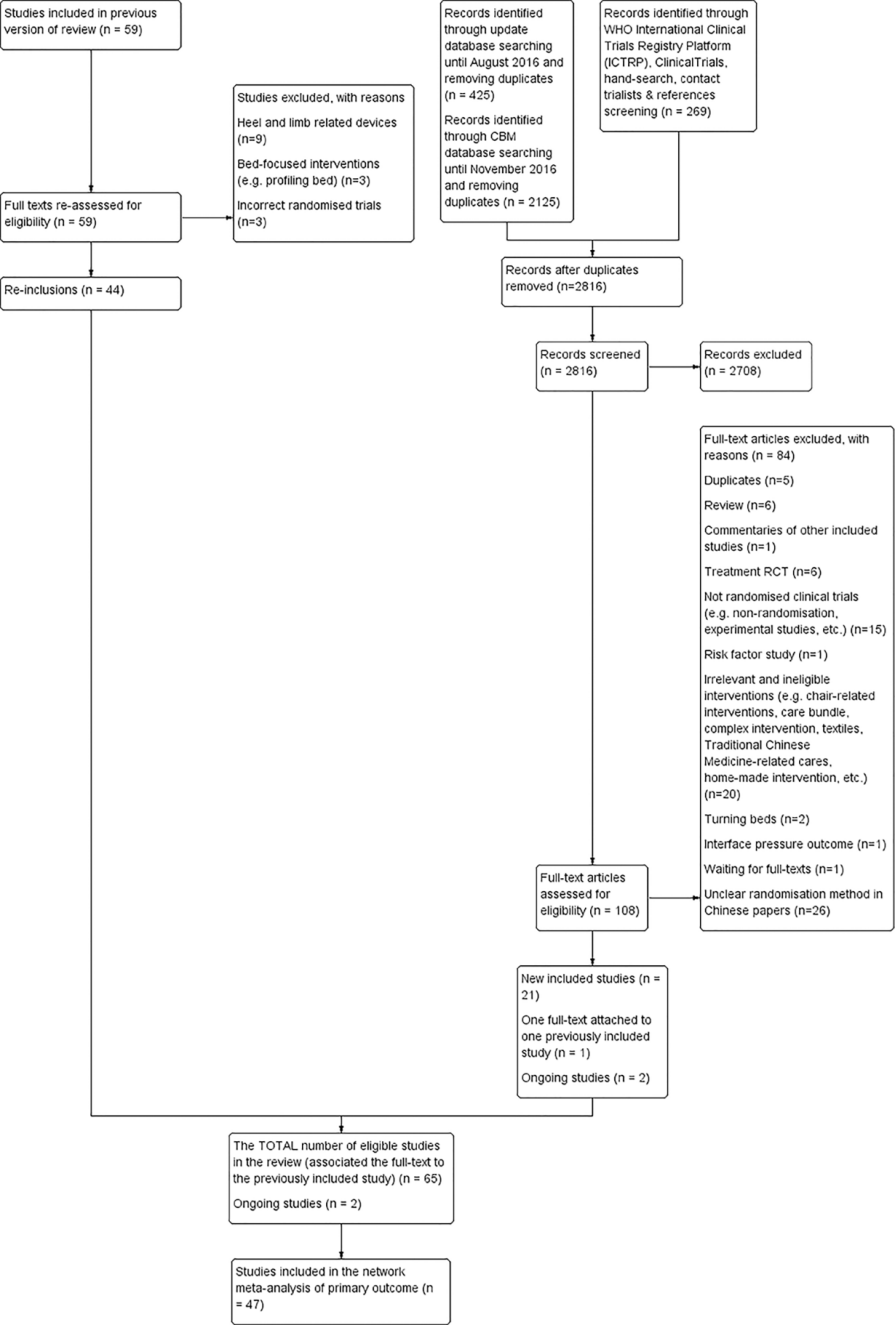
Flow diagram of included studies.

The search identified 2,816 records. Full-text screening of 108 potentially eligible studies led to inclusion of 22 studies; eight published in English (one of the eight was then associated with an included study in the McInnes and colleagues’ review [9]) and 14 studies published in Chinese. We also identified two on-going studies [41, 42]. In addition, our rescreening of the 59 studies included by McInnes and colleagues [9] identified 44 as specifically eligible for this review. In total therefore we included 65 studies in the review (see Fig 1, and S5 File for a reference list of included studies). Three were unpublished (one is a conference abstract [43] and two are research reports [44, 45].

### Trial and study population characteristics

The characteristics of included studies are summarised in Table 2. The 65 studies enrolled a total of 14,332 participants (median of study sample sizes: 100; range: 10 to 1,972). Setting was specified in 63 of 65 study reports (97%) and included accident and emergency departments and acute care, intensive care units, general medical wards, orthopaedic centres, operating theatres, and long-term care settings (i.e. nursing homes, extended care facilities, rehabilitation wards, long-term units).

**Table 2:** Characteristics of included studies.

Study (Reference numbers of included studies)	Country	Setting	Randomised number of participants (a priori calculation)	Participants (clear criteria); Age ^‡^ (years); Sex (male/female)	Baseline skin status	Comparisons (allocated numbers in arms)	Group interventions	Funding sources	Outcomes and follow up (days)	Comments
Andersen 1982 (Ref 1)	Denmark	Hospital in general	482 (Yes)	Acute conditions (Yes); Over 70 years on average; 206/276	No existing pressure ulcers; At risk	Alternating air mattress (166) vs water mattress (155) vs standard hospital mattress (161)	Powered active air surfaces vs non-powered reactive water surfaces vs standard hospital surfaces	Unclear	Incidence of pressure ulcers; Patient comfort; 10	Three-arm RCT
Aronovitch 1999 (Ref 2) *	USA	Operating theatre	217 (No)	Patients undergoing a surgery (> 4 hours anaesthesia) (Yes); 64.08±11.87; 156/58	No existing pressure ulcers; No high risk (Modified Norton Scale (MNS))	MicroPulse (112) vs Conventional management (105)	Powered active air surfaces vs standard hospital surfaces	Industry	Incidence of grade I to IV ulcers; 7	
Bennett 1998 (Ref 3)	USA	Various wards (i.e. two or more wards)	116 (No)	Patients incontinent of urine and/or faeces (Yes); Over 80 years on average; 45/71	Intact skin to grade II ulcer; High risk (Braden Scale)	Low-air-loss Hydrotherapy (58) vs Standard care (58)	Powered/non-powered reactive low-air-loss air surfaces vs standard hospital surfaces	Public & industry	Incidence of grade II to IV ulcers; 60	
Bliss 1967 (Ref 4)	UK	Hospital in general	83 (No)	General inpatients (Yes); 81.24; 27/56	Intact skin to grade II ulcer; At risk (MNS > 7)	Large-celled Ripple (42) vs Control (41)	Powered active air surfaces vs standard hospital surfaces	Public	Incidence of pressure ulcers; 14	
Cao 2013 (Ref 5) ^†^	China	Intensive care units	83 (No)	ICU elderly patients (Yes); 82.83±8.01; 68/15	No existing pressure ulcers; High risk (Braden < 12)	气垫床联合Action垫(translation: air mattress plus ACTION mattress) (43) vs 气垫床(translation: Air mattress) (40)	Unable to define vs unable to define	Unclear	Incidence of pressure ulcers; Unclear	
Cavicchioli 2007 (Ref 6)	Italy	Various wards	170 (No)	General patients (Yes); 77.51; 40/100	Intact skin to grade I ulcer; At risk (Braden scale)	Alternating pressure Duo 2 (86) vs Static mode Duo 2 (84)	Powered active air surfaces vs powered/non-powered reactive air surfaces	Unclear	Incidence of pressure ulcers; 14	
Chen 2015 (Ref 7)	China	Operating theatre	168 (No)	Patients undergoing surgeries with prone position (No); 39.6±21.7; 104/64	Unclear; Unclear risk	海绵垫和可固定式海绵体位垫 (translation: Foam pad) (112) vs 可固定式高分子凝胶垫(translation: Gel pad) (56)	Non-powered reactive gel surfaces vs non-powered reactive foam surfaces	Public	Incidence of pressure ulcers; Unclear	
Cobb 1997 (Ref 8)	USA	Various wards	123 (No)	General patients (Yes); Median 64; 70/53	No existing pressure ulcers; High risk (Braden scale)	Low air loss bed (62) vs Static air mattress overlay (61)	Powered/non-powered reactive low air loss surfaces vs powered/non-powered reactive air surfaces	Public	Incidence of pressure ulcers; 40	Research program report; unpublished
Collier 1996 (Ref 9) ^¶^	UK	General medical ward	90 (No)	General patients (No); Not reported (NR); 40/59	Unclear; Unclear risk	Seven foam mattresses (81) vs standard hospital mattress (9)	Non-powered reactive foam surfaces vs standard hospital surfaces	Unclear	Incidence of pressure ulcers; Unclear	
Conine 1990 (Ref 10)	Canada	Extended care facility	187 (No)	Chronic neurological diseases (Yes); 37.16±13.1; 60/88	No existing pressure ulcers; High risk (Norton scale)	Alternating-pressure overlay (93) vs Silicore^TM^ (Spenco) overlay (94)	Powered active air surfaces vs non-powered reactive fibre surfaces	Public	Incidence of grade I to IV pressure ulcers; 90	
Cooper 1998 (Ref 11) ^†^	UK	Orthopaedic	100 (No)	Emergency orthopaedic trauma (Yes); 83±7.65; 16/84	No existing pressure ulcers; At risk (Waterlow scale > 15)	Dry flotation mattress (Roho) (49) vs Dry flotation mattress (Sofflex) (51)	Powered/non-powered reactive air surfaces vs powered/non-powered reactive air surfaces	Industry	Incidence of grade I to IV pressure ulcers; Patient comfort; 7	
Daechsel 1985 (Ref 12)	Canada	Extended care facility	32 (No)	Chronic neurological conditions (Yes); 40.55±13.9; 16/16	No existing pressure ulcers; High risk	Alternating-pressure mattress (16) vs Silicore overlay (16)	Powered active air surfaces vs non-powered reactive fibre surfaces	Public & industry	Incidence of grade I to IV pressure ulcers; 90	
Demarre 2012 (Ref 13) ^†^	Belgium	Various wards	610 (Yes)	General inpatients (Yes); 76.33±14.02; 241/369	Intact skin to grade I ulcer; At risk (Braden scale)	Multi-staged alternating pressure air mattress (298) vs Single-staged alternating pressure air mattress (312)	Powered active air surfaces vs powered active air surfaces	Public & industry	Incidence of grade II to IV pressure ulcers; Patient comfort; 14	
Economides 1995 (Ref 14)	USA	Unclear	12 (No)	Grade 4 ulcers patients requiring myocutaneous flap closure (No); 40.5±16.29; 11/1	Grade IV ulcers	Air-fluidised Clinitron (6) vs Roho dry flotation mattress (6)	Powered reactive air-fluidised surfaces vs powered/non-powered reactive air surfaces	Unclear	Incidence of pressure ulcer (new wound breakdown); 14	
Ewing 1964 (Ref 15) ^¶^	Australia	Geriatric unit	36 (No)	General inpatients (No); 72.5 on average; NR	Unclear; Unclear risk	Sheepskins (18) vs control (18)	Non-powered reactive sheepskin surfaces vs standard hospital surfaces	Unclear	Incidence of pressure ulcers; 180	
Feuchtinger 2006 (Ref 16)	Germany	Operating theatre	175 (Yes)	Cardiac surgery patients (Yes); 67.79±10.9; 125/50	Intact skin to grade I ulcer; Unclear risk	Thermo (85) vs standard operating theatre configuration (90)	Non-powered reactive foam surfaces vs standard hospital surfaces	Unclear	Incidence of pressure ulcers; 5	
Finnegan 2008 (Ref 17) ^¶^	USA	Orthopaedic	40 (No)	Patients requiring tissue deficit repair (Yes); 55.45; 21/12	Ulcers	NIMBUS 3 (19) vs Air-fluidised bed (21)	Powered active air surfaces vs powered reactive air-fluidised surfaces	Industry	Incidence of pressure ulcers; Patient comfort; 8	
Gao 2014 (Ref 18)	China	Hospital in general	96 (No)	Persistent vegetative state patients (Yes); 41.38±3.25; 50/46	Unclear; Unclear risk	交替式气垫床(translation: Alternating pressure air mattress) (48) vs 普通床垫(translation: usual mattress) (48)	Powered active air surfaces vs standard hospital surfaces	Unclear	Incidence of pressure ulcers; Unclear	
Gray 1994 (Ref 19) ^¶^	UK	Various wards	170 (No)	General orthopaedic patents (Yes); 75.06±10.31; 66/104	No existing pressure ulcers; At risk (Norton scale)	Softfoam mattress (90) vs Standard hospital mattress (80)	Non-powered reactive foam surfaces vs standard hospital surfaces	Industry	Incidence of pressure ulcers; Patient comfort; 10	
Gray 1998 (Ref 20) ^†^ ^¶^	UK	Various wards	100 (No)	General inpatients (Yes); 65±5.88; 61/39	No existing pressure ulcers; At risk (Norton scale)	Transfoamwave (50) vs Transfoam (50)	Non-powered reactive foam surfaces vs non-powered reactive foam surfaces	Unclear	Incidence of pressure ulcers; Patient comfort; 10	
Gray 2008 (Ref 21)	UK	Acute care	100 (No)	General inpatients (No); 83.2; NR	Unclear; High risk (Norton scale)	Softform Premier Active (50) vs alternating pressure air mattress (50)	Powered hybrid air surfaces vs powered active air surfaces	Unclear	Incidence of pressure ulcers; Unclear	
Gunningberg 2000 (Ref 22)	Sweden	Accident and emergency department & wards	101 (Yes)	Suspected hip fracture patients (Yes); 85 (66 to 102); 81/20	No existing pressure ulcers; At risk (MNS <. 21)	Visco-elastic foam (48) vs Standard hospital mattress (53)	Non-powered reactive foam surfaces vs standard hospital surfaces	Industry	Incidence of pressure ulcers; 14	
Hampton 1997 (Ref 23) ^†^ ^¶^	UK	Unclear	75 (No)	Unclear (No); 77 on average; NR	Unclear; Unclear risk	Alternating-pressure (Cairwave) (36) vs Alternating-pressure (Airwave) (39)	Powered active air surfaces vs powered active air surfaces	Unclear	Incidence of pressure ulcers; 20	
Hofman 1994 (Ref 24)	Netherlands	Orthopaedic	46 (Yes)	Femoral-neck fracture patients (Yes); 84.42±7.52; 6/38	Intact skin to grade I ulcer; High risk (Dutch score)	Cubed foam (23) vs Standard hospital mattress (23)	Non-powered reactive foam surfaces vs standard hospital surfaces	Unclear	Incidence of pressure ulcers; 14	
Inman 1993 (Ref 25)	Canada	Intensive care units	100 (Yes)	Intensive care unit patients (Yes); 64.4±14.19; 51/47	Unclear; At risk	Low-air-loss (50) vs Standard intensive care unit bed (50)	Powered/non-powered reactive low-air-loss air surfaces vs standard hospital surfaces	Industry	Incidence of pressure ulcers; 17	
Ji 2011 (Ref 26)	China	Hospital in general	60 (No)	General patients (No); 72.3 (58 to 86); 53/37	No existing pressure ulcers; Unclear risk	交替式气垫床(translation: Alternating pressure air mattress) (30) vs 医院标准普通泡沫海绵床垫(translation: Standard hospital foam mattress) (30)	Powered active air surfaces vs standard hospital surfaces	Unclear	Incidence of pressure ulcers; 28	
Jiang 2015 (Ref 27)	China	Various wards	1074 (No)	Post-operative patients (Yes); 57.94±15.55; 621/453	Unclear; High risk (Braden score 13 on average)	动态空气床垫(translation: Alternating pressure air mattress) (512) vs 静态空气床垫(translation: Static air mattress) (562)	Powered active low-air-loss air surface vs powered/non-powered reactive air surfaces	Unclear	Incidence of pressure ulcers; Unclear	
Jolley 2004 (Ref 28)	Australia	Hospital in general	539 (Yes)	General inpatients (Yes); Mean 62.14 (range 18 to 99); 218/223	No existing pressure ulcers; Low to moderate risk (Braden scale)	Sheepskin (270) vs Usual care (269)	Non-powered reactive sheepskin surfaces vs standard hospital surfaces	Public & industry	Incidence of pressure ulcers; Unclear	
Kemp 1993 (Ref 29) ^†^	USA	Various wards	84 (No)	General inpatients (Yes); 81±8; 26/58	No existing pressure ulcers; At risk (Braden scale)	Convoluted foam (45) vs Solid foam (39)	Non-powered reactive foam surfaces vs non-powered reactive foam surfaces	Public & industry	Incidence of pressure ulcers; 30	
Laurent 1998 (Ref 30)	Belgium	Intensive care unit	312 (No)	Cardiovascular surgery patients (Yes); 64.0±11.88; 214/98	Unclear; Unclear risk	Nimbus and Tempur (constant low pressure) (77) vs Nimbus (alternating pressure) intensive care unit (80) vs Tempur (constant low pressure) postoperation (75) vs Standard mattress (80)	Powered active air surfaces plus non-powered reactive foam surfaces vs powered active air surfaces vs non-powered reactive foam surfaces vs standard hospital surfaces	Unclear	Incidence of pressure ulcers; Unclear	Conference abstract; unpublished; 2x2 factorial design
Lazzara 1991 (Ref 31)	USA	Nursing home	66 (No)	Nursing home residents (Yes); 83.61±8; 6/21	Ulcers; At risk	Air-filled (SofCare) overlay (33) vs Gel mattress (33)	Powered/non-powered reactive air surfaces vs non-powered reactive gel surfaces	Industry	Incidence of pressure ulcers; 180	
Liu 2012 (Ref 32)	China	Hospital in general	200 (No)	General patients (No); 60.14±2.35; 113/87	Unclear; At risk (Braden scale)	动态空气垫 (translation: Alternating pressure air mattress) (100) vs 静态空气垫(translation: Static air mattress) (100)	Powered active air surfaces vs powered/non-powered reactive air surfaces	Unclear	Incidence of pressure ulcers; Unclear	
Malbrain 2010 (Ref 33)	Belgium	Intensive care unit	16 (No)	ICU patients requiring mechanical ventilation (Yes); 64.2±15.99; 6/10	Ulcers; High risk (Norton scale)	NIMBUS 3 (8) vs ROHO DRY FLOATATION (8)	Powered active air surfaces vs powered/non-powered reactive air surfaces	Industry	Incidence of pressure ulcers; Unclear	
McGowan 2000 (Ref 34)	Australia	Orthopaedic	297 (Yes)	Orthopaedic patients (Yes); 73.79±7.88; 127/170	No existing pressure ulcers; Low to moderate risk (Braden scale)	Sheepskin (155) vs Control (142)	Non-powered reactive sheepskin surfaces vs standard hospital surfaces	Public & industry	Incidence of pressure ulcers; Unclear	
Mistiaen 2009 (Ref 35)	Netherlands	Nursing home	588 (Yes)	General residents (Yes); 78 (26 to 98); 183/405	Grade I; No risk (Braden score 18.2 on average)	Sheepskin (295) vs Usual care (293)	Non-powered reactive sheepskin surfaces vs standard hospital surfaces	Public	Incidence of pressure ulcers; 30	
Nixon 1998 (Ref 36)	UK	Operating theatre	446 (Yes)	Surgery patients (Yes); Over 55 years enrolled; 235/208	Intact skin to grade I ulcer; Unclear risk	Visco-elastic pad (222) vs Standard operating theatre mattress (224)	Non-powered reactive foam surfaces vs standard hospital surfaces	Public	Incidence of pressure ulcers; 8	
Nixon 2006 (Ref 37) ^†^	UK	Various wards	1972 (Yes)	General patients (Yes); 75.2±9.46; 711/1260	Intact skin to grade II ulcer; Unclear risk	Alternating-pressure overlay (990) vs Alternating-pressure mattress (982)	Powered active air surfaces vs powered active air surfaces	Public	Incidence of grade II to IV pressure ulcers; Patient comfort; 60	
Ozyurek 2015 (Ref 38) ^†^	Turkey	Intensive care unit	105 (No)	Intensive care unitpatients (Yes); 64.99±15.18; NR	Intact skin to grade I ulcer; At risk (Braden scale < 18)	Viscoelastic foam 1 (53) vs Viscoelastic foam 2 (52)	Non-powered reactive foam surfaces vs non-powered reactive foam surfaces	Public	Incidence of pressure ulcers; 7	
Price 1999 (Ref 39)	UK	Orthopaedic	80 (No)	Femoral neck fractured patients (No); Mean 82.2 (range 64.4 to 98.4); 16/64	Unclear; High risk (Medley score >25)	Repose (40) vs Nimbus II (40)	Powered/non-powered reactive air surfaces vs powered active air surfaces	Public & industry	Incidence of pressure ulcers; Patient comfort (score); 14	
Qu 2014 (Ref 40) ^¶^	China	Neurological units	90 (No)	General patients (Yes); 56.70±15.61; 63/27	Unclear; At risk (Braden scale < 16)	静态空气垫(translation: Static air mattress) (45) vs 动态空气垫 (translation: Alternating pressure low-air-loss air mattress) (45)	Non-powered reactive air surfaces vs powered active low-air-loss air surfaces	Unclear	Incidence of pressure ulcers; 14	
Rafter 2011 (Ref 41) ^†^ ^¶^	UK	Rehabilitation wards	10 (No)	General patients (Yes); 74.9; NR	Intact skin to grade II ulcer; High risk (Waterlow scale)	Dyna-Form (5) vs Softform Premier Active (5)	Powered hybrid air surfaces vs powered hybrid air surfaces	Industry	Incidence of pressure ulcers; Patient comfort; 28	
Ricci 2013 (Ref 42) ^†^ ^¶^	Italy	Long-term units	50 (No)	General inpatients (Yes); 84.7±7.76; 8/42	Intact skin to grade I ulcer; Moderate to high risk.(Braden scale)	Aiartex (25) vs Akton (25)	Non-powered reactive foam surfaces vs non-powered reactive foam surfaces	Industry	Incidence of pressure ulcers; Patient comfort; 28	
Russell 2000 (Ref 43)	Canada	Operating theatre	198 (No)	Cardiothoracic surgery patients (Yes); 65.2±10.75; 150/48	No existing pressure ulcers; Unclear risk	MicroPulse (98) vs Conventional care (100)	Powered active air surfaces vs standard hospital surfaces	Industry	Incidence of pressure ulcers; 7	
Russell 2003 (Ref 44)	UK	Various wards	1168 (Yes)	General patients (Yes); 83 (79 to 87); 391/777	Unclear; At risk (Waterlow scale)	CONFOR-Med (564) vs Standard mattress (604)	Non-powered reactive foam surfaces vs standard hospital surfaces	Industry	Incidence of pressure ulcers; Patient comfort (score); 11	
Sanada 2003 (Ref 45)	Japan	Acute care	108 (No)	Patients needing to be bed bound with head elevated (Yes); 71.26±12.32; 42/40	No existing pressure ulcers; At risk (Braden scale)	Double & single-layer air overlay (73) vs Standard hospital mattress (35)	Powered active air surfaces vs standard hospital surfaces	Unclear	Incidence of pressure ulcers; Unclear	
Santy 1994 (Ref 46)	UK	Orthopaedic	505 analysed (Yes)	Hip fracture patients (Yes); Mean 80.23 (range 78 to 81); NR	Intact skin to grade II ulcer; At risk (Waterlow scale)	Four foam mattresses (441) vs NHS Contract (64)	Non-powered reactive foam surfaces vs standard hospital surfaces	Public	Incidence of pressure ulcers; 12	Research report; unpublished
Schultz 1999 (Ref 47)	USA	Operating theatre	413 (Yes)	Surgery patients (Yes); 65.71±12.28; 266/147	No existing pressure ulcers; Unclear risk	Experimental mattress overlay (206) vs Usual care (207)	Non-powered reactive foam surfaces vs standard hospital surfaces	Public & industry	Incidence of pressure ulcers; 6	
Sideranko 1992 (Ref 48)	USA	Intensive care unit	57 (No)	Intensive care unit patients (Yes); 65.85±14.69; 33/24	No existing pressure ulcers; Unclear risk	Alternating air mattress (20) vs Static air mattress (20) vs Water mattress (17)	Powered active air surfaces vs powered/non-powered reactive air surfaces vs non-powered reactive water surfaces	Unclear	Incidence of pressure ulcers; Unclear	Three-arm RCT
Stapleton 1986 (Ref 49)	UK	Orthopaedic	100 (No)	Neck femur fractured patients (Yes); 81 on average; 0/81	No existing pressure ulcers; At risk (Norton score < 14)	Large Cell Ripple (32) vs Spenco pad (34) vs Polyether foam pad (34)	Powered active air surfaces vs non-powered reactive fibre surfaces vs non-powered reactive foam surfaces	Public	Incidence of pressure ulcers; Unclear	Three-arm RCT
Takala 1996 (Ref 50)	Finland	Intensive care unit	40 (No)	Intensive care unit patients (Yes); 61.42±14.32; 25/15	Unclear; Unclear risk	Carital Optima (21) vs Standard hospital foam mattress (19)	Powered/non-powered reactive air surfaces vs standard hospital surfaces	Industry	Incidence of pressure ulcers; 14	
Tang 2014 (Ref 51) ^†^	China	Intensive care unit	800 (No)	Intensive care unit patients (Yes); 72.0±2.1; 441/359	No existing pressure ulcers; High risk (Braden score < 12)	防压疮气垫床(translation: Anti-ulcer air mattress) (405) vs 理疗充气床垫辅以患者换床单(translation: Physiotherapy air mattress plus replacement sheets) (395)	Unable to define vs unable to define	Unclear	Incidence of pressure ulcers; Unclear	
Taylor 1999 (Ref 52) *	UK	Acute care setting	44 (Yes)	General inpatients (Yes); 68.38±3.11; 25/19	No existing pressure ulcers; At risk	Pegasus Trinova (22) vs Alternating pressure air mattress (22)	Powered hybrid air surfaces vs powered active air surfaces	Unclear	Incidence of pressure ulcers; Unclear	
Theaker 2005 (Ref 53)	UK	Intensive care unit	62 (Yes)	Intensive care unit patients (Yes); 65 (26 to 85); 39/23	No existing pressure ulcers; High risk	TheraPulse (30) vs Hill-Rom Duo (32)	Powered hybrid low air loss surfaces vs powered hybrid air surfaces	Unclear	Incidence of pressure ulcers; 14	
Vanderwee 2005 (Ref 54)	Belgium	Various wards	447 (Yes)	General patients (Yes); 81.5 (76 to 88); 164/283	Intact skin to grade I ulcer; At risk (Braden score < 17)	Alternating pressure air mattress (222) vs Visco-elastic foam mattress (225)	Powered active air surfaces vs non-powered reactive foam surfaces	Public & industry	Incidence of grade II to IV pressure ulcers; Unclear	
van Leen 2011 (Ref 55)	Netherlands	Nursing home	83 (Yes)	Nursing home residents (Yes); 82.09±8.18; 16/67	No existing pressure ulcers; High risk (Norton score < 12)	Static air overlay (42) vs Standard hospital mattress (41)	Powered/non-powered reactive air surfaces vs standard hospital surfaces	Unclear	Incidence of grade II to IV pressure ulcers; 180	
van Leen 2013 (Ref 56)	Netherlands	Nursing home	41 (No)	Nursing home residents (Yes); 79.97; 9/32	No existing pressure ulcers; Medium to high risk (Braden score 6 to 19)	Static air overlay (20) vs Viscoelastic foam mattress (21)	Powered/non-powered reactive air surfaces vs non-powered reactive foam surfaces	Unclear	Incidence of grade II to IV pressure ulcers; 180	Cross-over design (data at the first phase collected)
Vermette 2012 (Ref 57)	Canada	Various wards	110 (Yes)	General patients (Yes); 77.8±12.76; 44/66	No existing pressure ulcers; Moderate to very high risk (Braden score < 14)	Inflated static overlay (55) vs Microfluid static overlay (50)	Powered/non-powered reactive air surfaces vs non-powered reactive foam surfaces	No	Incidence of pressure ulcers; Patient comfort; 14	
Vyhlidal 1997 (Ref 58) ^†^	USA	Nursing homes	40 (No)	General patients (Yes); 77.2±14.21; 18/22	No existing pressure ulcers; At risk (Braden score < 18)	IRIS 3000 (20) vs MAXIFLOAT (20)	Non-powered reactive foam surfaces vs non-powered reactive foam surfaces	Industry	Incidence of pressure ulcers; 21	
Wang 2016 (Ref 59)	China	Intensive care unit	160 (No)	Cardiothoracic surgery patients (Yes); 53.54±11.28; 90/70	No existing pressure ulcers; At risk (Braden score < 16)	自动压力交替床垫 (translation: Automatically alternating pressure air mattress) (80) vs 交替式减压床垫(translation: Alternating pressure relieving air mattress) (80)	Powered hybrid air surfaces vs powered active air surfaces	Unclear	Incidence of pressure ulcers; Unclear	
Wei 2016 (Ref 60)	China	Intensive care unit	60 (No)	Coma patients (Yes); 69.80±8.35; NR	No existing pressure ulcers; High risk (Braden score < 12)	常规护理和气垫床(translation: Air surfaces plus usual cares) (30) vs 常规护理(translation: Usual cares) (30)	Powered active air surfaces vs standard hospital surfaces	Public	Incidence of pressure ulcers; Unclear	
Whitney 1984 (Ref 61)	USA	Hospital in general	51 (No)	General patients (No); 63.2 (19 to 91); NR	Existing skin breakdown; Unclear risk	Alternating-pressure mattress (25) vs Convoluted foam pad (26)	Powered active air surfaces vs non-powered reactive foam surfaces	Unclear	Incidence of pressure ulcers; 8	
Xu 2015 (Ref 62) ^†^	China	Hospital in general	76 (No)	General patients (Yes); 67.45±2.75; 41/35	No existing pressure ulcers; High risk (Braden score < 12)	喷气式防压疮垫(translation: Low-air-loss anti-ulcer air mattress (39) vs 电动防压疮垫(translation: Powered anti-ulcer air mattress) (37)	Powered active low-air-loss air surfaces vs powered active low-air-loss air surfaces	Public	Incidence of pressure ulcers; 14	
Zhao 2008 (Ref 63) ^†^	China	Hospital in general	46 (No)	General patients (No); 71.6 (38 to 91); 33/13	Unclear; Unclear risk	按摩式气垫床(translation: Massage air mattress) (25) vs 喷气式气垫床(translation: Low-air-loss air mattress) (21)	Powered active low-air-loss air surfaces vs unable to define	Unclear	Incidence of pressure ulcers; Unclear	
Zhan 2014 (Ref 64) ^†^	China	Orthopaedic	64 (No)	Pelvic fracture (Yes); 48.17±8.23; 35/29	No existing pressure ulcers; High risk (Braden score < 12)	冷疗气垫疗法(translation: Water cushion in addition to air mattress) (32) vs 气垫床(translation: Air mattress) (32)	Unable to define vs unable to define	Public	Incidence of pressure ulcers; Unclear	
Zhang 2015 (Ref 65)	China	Intensive care unit	158 (No)	Intensive care unit patients (Yes); 56.99±14.55; 104/52	No existing pressure ulcers; At risk (Braden score < 16)	气垫床(translation: Air mattress (78) vs 凝胶海绵床垫(translation: Gel mattress) (78)	Powered active low-air-loss air surfaces vs non-powered reactive gel surfaces	Public	Incidence of pressure ulcers; Unclear	

* studies that did provide data on the incidence proportion of pressure ulcers but did not report the numbers of analysed participants or drop-outs and thus were included in sensitivity analysis solely

† Studies that compared a support surface with another from the same intervention groups or which evaluated support surfaces without sufficient information to define their intervention group; these studies were not included in the network meta-analysis because the interventions were not linked into the network.

¶ Studies that did not present numbers of events, or reported zero events in both arms and thus were excluded from network meta-analysis

‡ Age was presented as mean ± SDs, median (range), or median/mean where available.

The average age of participants was specified for 64 studies (98%) and ranged from 37 to 85 years (median: 70 years). Gender was specified for 57 studies (with 13,158 participants), within these 53% of participants were female. Forty included studies (62%) recruited only participants with intact skin at baseline and/or those with grade I ulcers. Ten studies (15%) enrolled participants with existing ulcers (recorded or assumed to be grade II or above). In the 44 studies (68%) that clearly stated duration of follow-up the median was 14 days (range: 5 to 180). There were 23 studies (35%) that were completely or partly funded by industry and 15 studies (23%) supported by public funding.

In terms of intervention groups, of 65 studies, four (6%) used support surfaces that were impossible to classify into an intervention group due to insufficient detail; and an additional 11 studies (17%) compared support surfaces within the same intervention groups (see Table 2). These 15 studies were removed from quantitative analysis because their intervention groups were unconnected to any network although they are still included in the review.

### Risk of bias assessment

Of 65 studies, 28 studies (43%) were judged to have no serious limitations; and the remaining 37 studies (57%) had serious or very serious limitations (see S6 File).

### Network meta-analysis

We conducted two main network meta-analyses; the first for pressure ulcer incidence (the Prevention Network) and the second for patient comfort (the Comfort Network). No network was formed for time-to- pressure ulcer incidence because the eight (12%) studies (Refs 13, 28, 34, 35, 37, 53, 54, 58 in the Table 2) with available outcome data did not form a network connecting more than two intervention groups.

#### Prevention network: Summary of included evidence

All 65 included studies reported the outcome of pressure ulcer incidence, of which 20 were excluded from analysis: three reporting zero events in both arms (Refs 9, 17, 40 in the Table 2) (see Discussion for further consideration of these three studies), six with incomplete outcome data and intervention descriptions (Refs 5, 15, 19, 51, 63, 64 in the Table 2), and 11 comparing support surfaces from the same intervention groups (Refs 11, 13, 20, 23, 29, 37, 38, 41, 42, 58, 62 in the Table 2) (see Table 2). Of the remaining 45 studies, 43 were included in the main analysis and two (Refs 2 and 52 in the Table 2) were only considered in the sensitivity analysis imputing missing data. The 43 studies (Ref 1, 3, 4, 6–8, 10, 12, 14, 16, 18, 21, 22, 24–28, 30–36, 39, 43–50, 53–57, 59–61, 65 in Table 2), involved 9,430 participants and formed 24 direct comparisons and a network of 14 intervention groups.

#### Prevention network: Main findings

**Fig 2:**
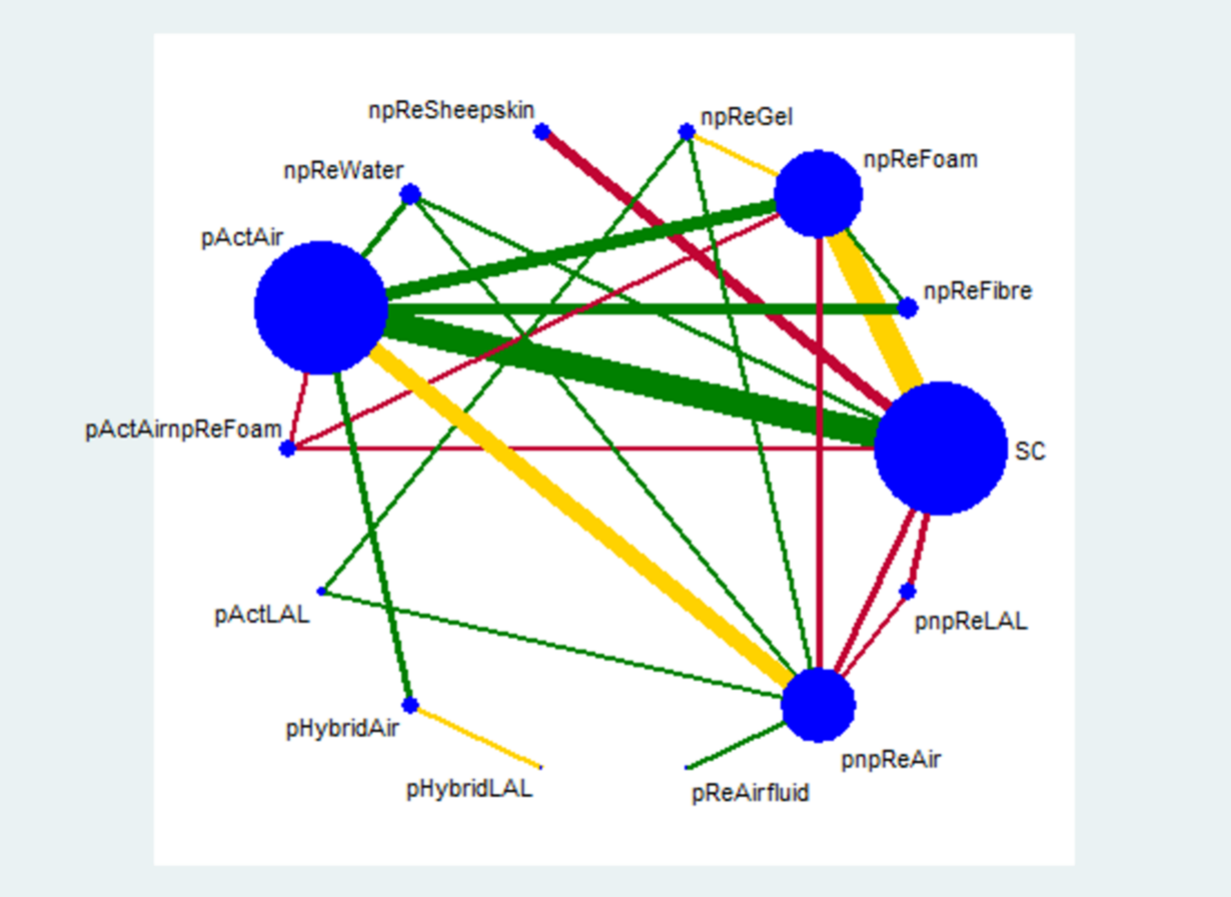
Network plot for the incidence of pressure ulcers produced by STATA *networkplot* command. Fourteen intervention groups are coded in the plot (i.e., nodes): SC = standard hospital surfaces, npReFibre = non-powered reactive fibre surfaces, npReFoam = non-powered reactive foam surfaces, npReGel = non-powered reactive gel surfaces, npReSheepskin = non-powered reactive sheepskin surfaces, npReWater = non-powered reactive water surfaces, pActAir = powered active air-cells surfaces, pActAirnpReFoam = powered active air-cells surfaces plus non-powered reactive foam surfaces, pActLAL = powered active low-air-loss air surfaces, pHybridAir = powered hybrid air-cells surfaces, pHybridLAL = powered hybrid low-air-loss air surfaces, pReAirfluid = powered reactive air-fluidised surfaces, pnpReAir = powered or non-powered reactive air-cells surfaces, and pnpReLAL = powered or non-powered reactive low-air-loss air surfaces. Each node size is proportional to the number of direct comparisons involving each intervention group. Taking any two of the six nodes forms 91 network contrasts. 24 lines between nodes represent direct comparisons driven by RCTs; and line thickness is proportional to the number of studies involved in each direct comparison. Direct evidence of two or more comparisons can generate indirect evidence for contrasts that did not involve a head-to-head RCT (e.g., indirect evidence for the comparison of npReFoam vs. npReWater generated from comparisons, for example, of npReFoam vs. SC and npReWater vs. SC). In this way, indirect evidence informs the remaining 67 of the 91 network contrasts. The risk of bias assessment was based on the most frequent level of bias recorded for studies included in that comparison and denoted using coloured lines (or links). A green link indicates no serious study limitations; yellow indicates serious limitations; and red indicates very serious limitations.

**Fig 3:**
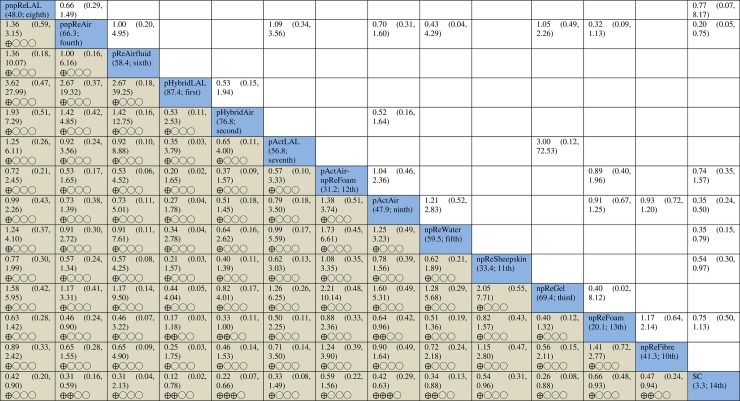
Results of pairwise meta-analyses via RevMan and network meta-analysis with consistency model via STATA for pressure ulcer incidence. Results of pairwise meta-analyses with the numbers of included studies and participants are presented above the diagonal cells (see S7 File); network meta-analysis results and the corresponding certainty of evidence assessments are shown below the diagonal cells. The diagonal cells show the codes of intervention groups and their SUCRA values and rankings in brackets: SC = standard hospital surfaces, npReFibre = non-powered reactive fibre surfaces, npReFoam = non-powered reactive foam surfaces, npReGel = non-powered reactive gel surfaces, npReSheepskin = non-powered reactive sheepskin surfaces, npReWater = non-powered reactive water surfaces, pActAir = powered active air-cells surfaces, pActAirnpReFoam = powered active air-cells surfaces plus non-powered reactive foam surfaces, pActLAL = powered active low-air-loss air surfaces, pHybridAir = powered hybrid air-cells surfaces, pHybridLAL = powered hybrid low-air-loss air surfaces, pReAirfluid = powered reactive air-fluidised surfaces, pnpReAir = powered or non-powered reactive air-cells surfaces, and pnpReLAL = powered or non-powered reactive low-air-loss air surfaces. ⨁⨁⨁◯ = Moderate certainty of evidence; ⨁⨁◯◯ = Low certainty of evidence; and ⨁◯◯◯ = Very low certainty of evidence.

The results of the pairwise and network meta-analyses are summarised in Fig 3 along with the GRADE certainty of evidence assessment for the network meta-analysis (see S7 File for pairwise meta-analyses; see S9 File for GRADE assessment). Of the 24 direct comparisons, 12 (50%) were judged to have serious or very serious limitations (see Fig 2). The entire network was considered to have serious study limitations. Additionally, the network was considered to be sparse as 13 of the 24 direct links were only informed by one study in each case.

The analysis results suggest that powered hybrid low-air-loss air surfaces have the highest probability of being the most effective intervention (SUCRA = 87.4%). However we remain uncertain as to the true ranking of these treatments because the certainty of evidence was very low (see Fig 3 and S9 File).

Overall, the evidence regarding the relative effects of support surfaces on pressure ulcer development is of low or very low certainty for 89 of the 91 network contrasts in the network. We present a further narrative summary of the network meta-analysis findings for what are considered key comparisons: the 13 intervention groups compared with standard hospital surfaces.

There is moderate certainty evidence that powered active air surfaces and powered hybrid air surfaces probably reduce the incidence of pressure ulcers compared with standard hospital surfaces (the latter having an assumed baseline risk of 219 per 1,000 participants) (RR 0.42, 95% CI 0.29 to 0.63; and RR 0.22, 95% CI 0.07 to 0.66, respectively). This represents 127 fewer people developing new ulcers per 1,000 (95% CI 81 to 155 per 1000) on powered active air surfaces and 171 fewer people developing new ulcers per 1,000 (95% CI 74 to 204) on powered hybrid air surfaces than on standard hospital surfaces. There is low-certainty evidence that non-powered reactive fibre surfaces, non-powered reactive water surfaces, powered hybrid low-air-loss air surfaces, and powered/non-powered reactive air surfaces may reduce pressure ulcer incidence compared with standard hospital surfaces. It is uncertain whether the remaining seven intervention groups reduce the incidence of pressure ulcers compared with standard hospital surfaces as the evidence is of very low certainty.

#### Prevention network: Results of transitivity assessment and heterogeneity analyses

We deemed that the transitivity assumption held and there was no suggestion of global inconsistency in the network using either the design-by-treatment interaction model or the model of Lu and Ades [34]. There was one loop with potential inconsistency (SC-npReFoam-pActAir): this was likely due to the influence of one pairwise meta-analysis in the loop which had high heterogeneity (non-powered reactive foam surfaces versus standard hospital surfaces).

The common network heterogeneity was moderate: tau^2^ = 0.195; and I^2^ = 56% (95% CI: 36 to 70%). This means that there was moderate variation in the mean effect size estimate across studies in each network contrast (i.e. in one network contrast, some included studies may suggest benefit for one intervention group but others may suggest harm). This moderate common network heterogeneity may be due to the very high heterogeneity (I^2^ > 75%) of three pairwise meta-analyses in the network (powered or non-powered reactive low-air-loss air surfaces, non-powered reactive sheepskin surfaces, and non-powered reactive foam surfaces compared with standard hospital surfaces). Additionally, subgroup analysis suggested that funding sources, considering operating theatres as settings or not, follow-up duration, and baseline skin status defined by authors may explain the network heterogeneity (tau^2^ from 0.195 to 0.160, 0.160, 0.178, and 0.129, respectively) but risk of bias assessment and setting may not (see S10 and S11 Files for the above analyses).

#### Prevention network: Results of sensitivity analyses

Sensitivity analyses did not suggest that missing data and unpublished data would affect the relative effects and rankings of interventions groups (see S8 File).

#### Prevention network: Publication bias

No funnel plot was produced for the pairwise meta-analyses because none included more than 10 studies. For the network meta-analysis, the comparison-adjusted funnel plot appeared slightly asymmetric, suggesting the possible presence of small-study effects; i.e. advanced support surfaces like powered hybrid air surfaces appear to have favourable prevention effects in small studies (see S9 File).

#### Comfort network: Summary of included evidence

**Fig 4:**
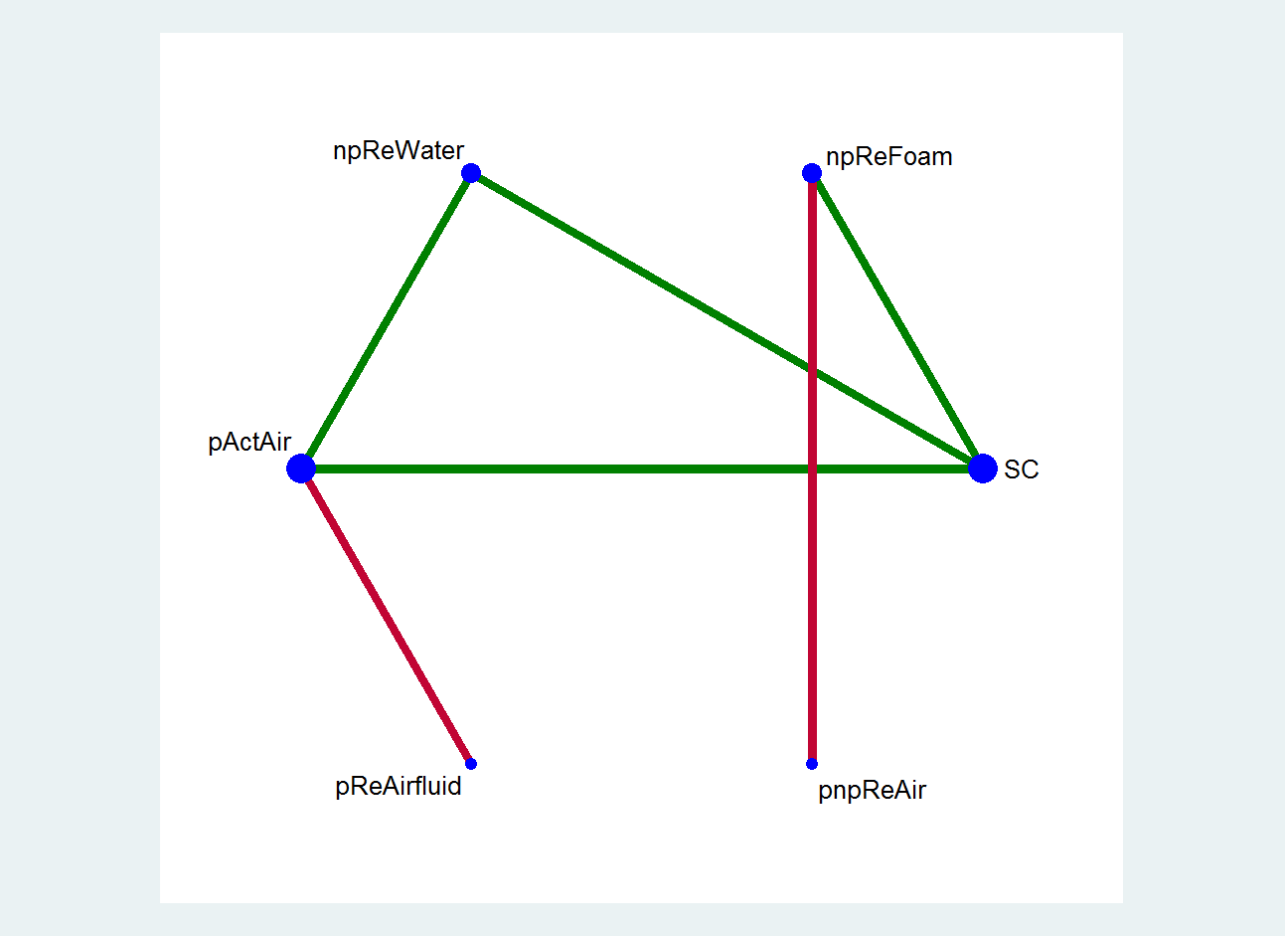
Network plot for the patient comfort on a support surface produced by STATA *networkplot* command. Six intervention groups (i.e., six nodes) are coded in the plot: SC = standard hospital surfaces, npReFoam = non-powered reactive foam surfaces, npReWater = non-powered reactive water surfaces, pActAir = powered active air-cells surfaces, pReAirfluid = powered reactive air-fluidised surfaces, pnpReAir = powered or non-powered reactive air-cells surfaces. Taking any two of the six nodes forms 15 network contrasts. The size of each node is proportional to the number of direct comparisons involving each intervention group. The six lines between nodes in the plot represent the only direct comparisons and line thickness is proportional to the number of studies involved in each direct comparison. The direct evidence arising from two or more comparisons can generate indirect evidence for contrasts that have not been compared in head-to-head RCTs (e.g., indirect evidence for the comparison of npReFoam vs. npReWater generated from comparisons of npReFoam vs. SC and npReWater vs. SC). In this way, indirect evidence informs nine of the 15 network contrasts. The risk of bias assessment was based on the most frequent level of bias recorded for studies included in that comparison and denoted using coloured lines (or links). A green link indicates no serious study limitation, yellow indicates serious limitations; and red very serious limitations.

Twelve of 65 studies (18%) presented outcome data on patient comfort, of which eight studies could not be included in the network: six studies were excluded as they compared support surfaces from the same intervention groups (Refs 11, 13, 20, 37, 41, 42 in the Table 2), and two (Refs 39, 44 in the Table 2) could not be connected to the network. Thus, the final network included four studies (Refs 1, 17, 19, 57 in the Table 2) (with 802 participants) which formed six direct comparisons and a network of six intervention groups (Fig 4).

#### Comfort network: Main findings

**Fig 5:**
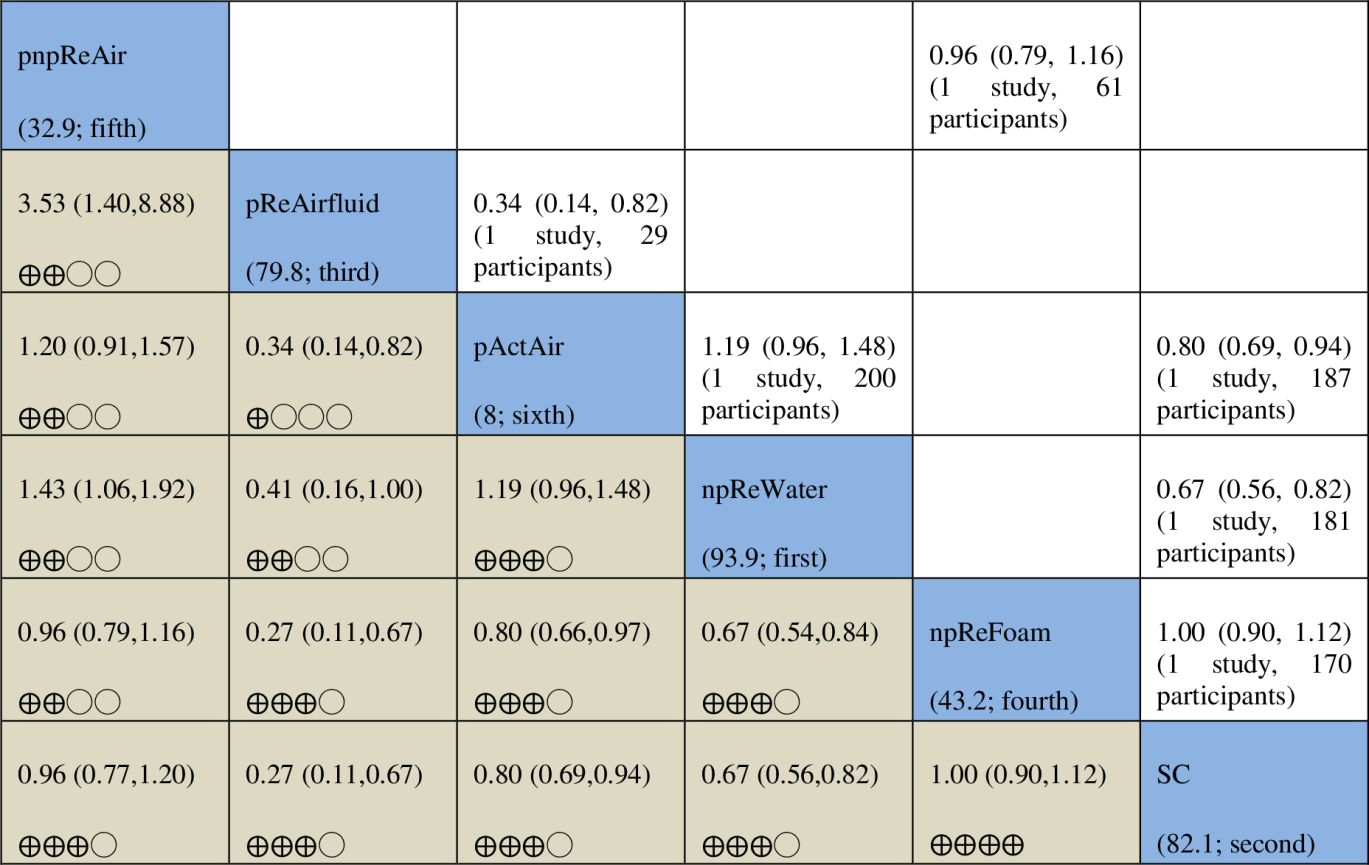
Results of pairwise meta-analyses and network meta-analysis with consistency model for the comfort of a support surface. Results of pairwise meta-analyses with the numbers of included studies and participants are presented above the diagonal cells; network meta-analysis results and the corresponding certainty of evidence assessments are shown below the diagonal cells. The diagonal cells show the codes of intervention groups and their SUCRA values and rankings in brackets: SC = standard hospital surfaces, npReFoam = non-powered reactive foam surfaces, npReWater = non-powered reactive water surfaces, pActAir = powered active air-cells surfaces, pReAirfluid = powered reactive air-fluidised surfaces, pnpReAir = powered or non-powered reactive air-cells surfaces. ⨁⨁⨁⨁ = High certainty of evidence; ⨁⨁⨁◯ = moderate certainty of evidence; ⨁⨁◯◯ = low certainty of evidence; and ⨁◯◯◯ = very low certainty of evidence.

The results of the pairwise and network meta-analyses are summarised in Fig 5 along with the GRADE-based assessment of the certainty of the evidence in the network meta-analysis. Four out of six (67%) direct comparisons had no serious limitations but another two had very serious limitations; and the whole network had serious limitations. The network was also sparse, each of the six direct comparisons was informed by only one study. It was not possible to explore publication bias because only four studies were included in the network.

Non-powered reactive water surfaces have the highest probability of being the best intervention in terms of comfort (SUCRA = 93.9%), and powered active air surfaces had the lowest probability of being the most comfortable (moderate certainty evidence) (see Fig 5).

Overall, evidence regarding the comfort of different surfaces is moderate or high certainty for nine of the fifteen network contrasts in the network but is low or very low for the remaining six contrasts. We present a summary here of key network meta-analysis findings for five intervention groups compared with standard hospital surfaces for the outcome of comfort.

Compared with standard hospital surfaces (with 866 per 1,000 participants reporting comfort on a support surface), powered active air surfaces, powered reactive water surfaces, and powered reactive air fluidised surfaces are probably less comfortable (RR 0.80, 95% CI 0.69 to 0.94; RR 0.67, 95% CI 0.56 to 0.82; and RR 0.27, 95% CI 0.11 to 0.67, respectively), corresponding to 173 fewer participants reporting comfort per 1,000 (95% CI 52 to 268), 286 fewer per 1,000 (95% CI 156 to 381), and 632 fewer per 1,000 (95% CI 286 to 771), respectively. Evidence for the three comparisons is of moderate certainty. There appears to be no difference in comfort between non-powered reactive foam surfaces and standard hospital surfaces (RR for participants reporting comfort 1.00, 95% CI 0.90 to 1.12; high-certainty evidence), nor between powered/non-powered reactive air surfaces and standard hospital surfaces (RR 0.96, 95% CI 0.77 to 1.20; moderate-certainty evidence).

#### Comfort network: Results of heterogeneity analyses

The entire network has a tau^2^ less than 0.001 suggesting no inconsistency or heterogeneity.

## Discussion

### Main findings

We present a systematic review and network meta-analysis of the evidence from 65 RCTs (with 14,332 participants) of the relative effects of different types of support surface in terms of pressure ulcer prevention and patient comfort. The specific support surfaces were successfully classified into 14 groups using an established classification system [4]. The included studies form two sparse networks; the studies were heterogeneous in terms of settings, participants’ baseline skin status, and follow-up durations; and over half of the studies had serious or very serious study limitations. All these issues reflect the uncertainty of evidence and the limited data included in each network.

There is moderate certainty evidence that powered active air surfaces and powered hybrid air surfaces probably reduce the risk of pressure ulceration compared with standard hospital mattresses; however participants were less likely to find powered active air-surfaces comfortable compared with standard hospital surfaces. Overall, it is highly uncertain which one of 14 classes of support surface is the most effective for preventing pressure ulcers but there is moderate certainty evidence that non-powered reactive water surfaces are probably the most comfortable of those surfaces compared. However, we identified only four studies (addressing six intervention groups) that assessed comfort and thus cannot link relevant findings to those of pressure ulcer prevention effects for all 14 intervention groups.

### Generalisability of results

The included studies were conducted in a variety of settings, and recruited participants with differing baseline skin status (the majority included people with intact skin and up to grade 1 ulceration). Most study participants were over 55 years old. It is worth noting that we assumed no prevention effect difference between mattresses and overlays with the same pressure redistribution mode and construction material (e.g. powered active air mattress vs. powered active air overlay) [46]. Rather, we used random-effects model to estimate on average relative effects of intervention groups [25]. So evidence in the review presents average relative effects that are generally applicable for the older adult population regardless of settings and baseline skin status.

Included studies also had a wide range of follow-up periods (ranging from 5 to 180 days with a median of 14). Based on available data we assumed no change in the rate of pressure ulcer incidence over time, and thus deemed that evidence on the relative effects is applicable for the case of an expected 14-day hospitalisation. We were unable to adjust the effects of interventions for follow-up duration or form a network of time-to-event data to understand how the hazard of ulcer development might change over time.

We identified only six studies from the operating theatre setting and thus the results of this review might not apply to operating theatre settings. Furthermore powered hybrid air surfaces were only evaluated in three small RCTs, and these studies were mainly conducted in ICU and acute care settings. Water-filled support surfaces were only evaluated in pre-1992 studies [47, 48] and these surfaces (and the evidence from these evaluations) might not be relevant today.

To assess the certainty of evidence for publication bias Salanti and colleagues [24] suggest not solely relying on evidence of funnel plots but also considering the scope of the literature search and the volume of unpublished data located. In this review, a comprehensive search was performed and unpublished data were also included where possible. All pairwise meta-analyses included fewer than 10 studies in each case; so for network contrasts, we did not assess funnel plots for publication bias and did not downgrade the certainty for this reason. For the ranking of intervention groups, though the asymmetric comparison-adjusted funnel plot suggested the possible presence of small-study effects in the overall network, given the comprehensive search and the inclusion of unpublished data but then the small number of included studies, we did not consider the asymmetry as concrete evidence of publication bias and thus did not downgrade the certainty of evidence for publication bias for this reason [24].

Finally, evidence on patient comfort should be also treated with caution because only a sub-set of surfaces (standard hospital surfaces, non-powered reactive foam surfaces, non-powered reactive water surfaces, powered active air-cells surfaces, powered reactive air-fluidised surfaces, and powered or non-powered reactive air-cells surfaces) were evaluated.

### Overall quality of the evidence

The certainty of evidence in this review was mainly downgraded for study limitations, imprecision and inconsistency.

In assessing study limitations, we acknowledge that blinding of participants and personnel (to protect against performance bias) is impractical for some comparisons (e.g., powered active air surfaces versus standard hospital surfaces) but could be ensured for others (e.g., powered active low-air-loss surfaces versus powered active air surfaces). Yet these practical issues do not change the importance of assessing the risk of performance bias which remains because, for example, caregivers’ knowledge of which support surface was provided might result in the imbalanced implementations of other co-interventions (e.g., repositioning) between study arms. Because of this we considered a consistent performance bias assessment across included studies in this network meta-analysis. We also considered that unblinded outcome assessment could substantially bias effect estimates (unblinded assessment has been found to exaggerate odds ratios by 36% for subjective binary outcomes) [49]. Downgrading for detection bias was undertaken on a study by study basis where blinded outcome assessment e.g., masked adjudication of photographs of pressure areas had not been utilised [50]. Most of the included studies (57%) were judged at serious or very serious limitations; reflected in the certainty of evidence assessment by downgrading once. We also considered risk of bias as a modifier in subgroup analysis to evaluate its impact on relative effects with no major impact detected. This finding is consistent with the first network meta-epidemiological study investigating the impact of risk of bias on relative effects [51]. Because of this we did not adjust relative effects for study limitations in any further analyses.

Secondly, as with pairwise meta-analysis, network meta-analysis is dependent on the volume of the included data and when data are sparse the confidence intervals around effect sizes are wide [52]. In this review, data in two networks were sparse; classifying support surfaces into intervention groups did not improve the sparseness. Most of the network contrasts had wide or very wide confidence intervals (see Fig 3), for which we downgraded the certainty of the evidence for imprecision. However, we could not tell to what extent imprecise effect sizes were related to sparseness or the use of the random-effects model (incorporating heterogeneity in effects). Therefore, although statistical approaches for addressing sparse networks have been proposed [52], there is no consensus as to the best approach and we did not apply them in this review.

Finally, the inconsistency assessment should be based on joint assessment of statistical heterogeneity and network inconsistency [24]. However, our decision to downgrade the certainty of evidence for inconsistency was largely based on the presence of moderate common heterogeneity (I^2^ = 56%) and not the network inconsistency. We did not find evidence of a global inconsistency by using both the design-by-treatment interaction model and the model of Lu and Ades [34]. There was one loop of linked data that was potentially inconsistent (i.e. SC-npReFoam-pActAir) but it was not clear whether this was truly inconsistent (based on statistical testing) or the result of high heterogeneity in some pairwise comparisons analyses [33] (see S10 File).

### Strengths and limitations

This work has a number of strengths. Firstly, we conducted a robust systematic review and searched for and included all eligible studies. For example, we sought Chinese studies and scrutinised them for evidence of robust randomisation. Then, to tackle the complex range of support surfaces available, we applied a “clinically meaningful elements” approach [20] using the NPUAP support surface classification system [4]. In this approach, we considered that the support surfaces with similar pressure redistribution modes, construction and function characteristics were associated with similar pressure ulcer prevention effects and treated as a “class” [20]. Alternative approaches included the “components and dismantling” approach, which considers these elements as independent components [20] or a different “lumping” approach grouping support surfaces with similar pressure redistribution modes (but different construction materials) together. The advantage of our approach was that it is coherent with the fact that support surfaces work, as combinations of some dependent but different elements, to prevent pressure ulcer development.

However, this review also has limitations. Firstly, in defining intervention groups, we disregarded co-interventions of included studies (e.g. repositioning) because these co-interventions were assumed to have been provided equally to participants in each trial arm (where the study had a pre-specified objective of comparing different support surfaces). It was often challenging to consider these co-interventions in more detail because some of the included studies regarded co-interventions as “usual care” but did not fully describe them.

Secondly “standard hospital surfaces” vary over time, by country and by setting [53] therefore the grouping “standard hospital surfaces” as the reference in network meta-analysis might bias the calculation of relative effects for other intervention groups across studies. However, because we considered standard hospital surfaces as a group and then estimated the average effect of the group, we did not overemphasise variations in “standard hospital surfaces” in the review.

Thirdly, we excluded three studies (Refs 9, 17, 40 in the Table 2) with zero events in both arms from the analysis because we were unable to analyse them within STATA and they were regarded as not contributing evidence of relative effects [54]. However, in practice, zero events in both arms could suggest that: (1) during the study process, both specific support surfaces of a study successfully reduced the risk of pressure ulcer development; and/or (2) because of a small sample size and short follow-up duration, a study is under-powered to show the potential pressure ulcer incidence in study arms. In either case zero events in both arms might be considered as “no statistical difference”, which is consistent with evidence from other studies that provided data.

Fourthly, we have to acknowledge that the inclusion of a multi-arm trial in the network meta-analysis may result in dependent effect estimates of comparisons within the trial so within-study correlation should have been addressed. However, methods for within-study correlation are less well developed than those for between-study correlation [55]. Besides, using current STATA mvmeta and network commands commonly assumes the within-study variance as known and ignores the within-study correlation [28, 56]. Hence we did not consider this issue in this network meta-analysis, which is consistent with common practice [55]. Finally, we found that 36% of included studies were funded completely or partly by industry; a finding that is consistent with the proportion of industry-funded studies across wound care (41%) [2] and funding sources may explain the network heterogeneity to some extent. However, we did not adjust relative effects for funding sources due to the limited number of included studies.

### Placing the findings in context of previous work

The earlier Cochrane review [9] reported that non-powered reactive foam surfaces reduce the proportion of participants developing a new ulcer compared with standard hospital surfaces. However, when we considered the certainty of evidence here we concluded that this result is highly uncertain. This has potential implications since popular pressure ulcer prevention guidelines currently recommend the use of “a high-specification foam mattress or foam theatre mattress” (i.e. non-powered reactive foam surfaces, in our review) for hospitalised adults at high risk [1, 53]; and non-powered, reactive, foam surfaces are in widespread clinical use (e.g., used by 48% of people at high risk [57]) and might have been used as standard support surfaces [53].

Regarding other reactive surfaces, McInnes and colleagues [9] presented separate analyses for static air-cells, water-, gel-, and fibre-filled surfaces but only evaluated direct, head-to-head comparisons and did not compare them with each other. In this review we defined these “constant low-pressure supports” as different intervention groups and compared them with each other in a network. Our network results suggest that the evidence is uncertain due to very low certainty for almost all network contrasts between these intervention groups. Two previous reviews [9, 10] evaluated the prevention effect of non-powered reactive sheepskin surfaces (a surface primarily used in the Australian context) compared with standard hospital surfaces, and concluded that the sheepskin surfaces are effective in reducing the proportion of participants developing a new ulcer. We regard this result as highly uncertain due to the low certainty of the evidence (downgraded for study limitations, imprecision, and inconsistency). Finally, considering that non-powered reactive foam surfaces are widely used and powered active air surfaces were suggested as effective in our analysis, we believe that RCTs of non-powered reactive foam surfaces compared with powered active air surfaces are urgently needed. We expect that an ongoing study that is planning to recruit 2,954 high-risk participants will help to reduce this evidence gap [42].

## Conclusions

Current moderate-certainty evidence from the prevention network suggests that, compared with standard hospital surfaces, powered active air surfaces and powered hybrid air surfaces probably reduce the incidence of pressure ulcers by 58% and 78% on average, respectively. However, a limited network for the outcome of comfort suggests lower comfort on powered active air surfaces than standard hospital surfaces. The evidence is uncertain for the pressure ulcer prevention effects of other intervention groups.

The network, with sparse data and very low quality of studies, suggests that more high-quality research is required. In particular more RCT evidence is required for powered hybrid air surfaces which were evaluated in only three studies in the network meta-analysis and non-powered reactive foam surfaces (which are widely used) and non-powered reactive sheepskins should be prioritised for research.

The poor quality of the existing evidence makes it particularly important that researchers undertaking any new research ensure study rigour. For example, it may be possible to minimise detection bias by using digital photography and adjudicators of the photographs being masked to support surface [50]. The existing studies are marred by short durations of follow up and we would recommend follow-up for at least 14 days or longer (e.g. 30 days) considering that most pressure ulcers occur in the first two to four weeks after admission [58]. Trialists should fully describe co-interventions (e.g. repositioning) and, if relevant, standard hospital surfaces as control arm, and report time-to-event data and ideally provide cost-effectiveness evidence. Additionally, the public sector should be encouraged to invest in further studies.

## Supporting information

S1 File The PRISMA network meta-analysis checklist. (DOCX)

S2 File Ovid MEDLINE Search Strategy. (DOCX)

S3 File The detailed steps to define and classify support surfaces. (DOCX)

S4 File Detailed procedures in network meta-analysis with STATA commands. (DOCX)

S5 File Reference list of included studies. (DOCX)

S6 File Risk of bias summary. (DOCX)

S7 File Results of pairwise meta-analyses for incidence of pressure ulcers. (PNG)

S8 File Sensitivity analysis of missing data and unpublished data imputation. (DOCX)

S9 File Quality of evidence assessment for the proportion of participants developing a new ulcer. (DOCX)

S10 File Heterogeneity and inconsistency assessment in the network meta-analysis. (DOCX)

S11 File Subgroup analyses. (DOCX)
