# Supplementary material for: Support surfaces for pressure ulcer prevention: A network meta-analysis
Source: PLoS One. 2018 Feb 23;13(2):e0192707. doi: 10.1371/journal.pone.0192707 (PMC5825032; doi:10.1371/journal.pone.0192707)
Supplement: S2 File — (DOCX) [file pone.0192707.s002.docx]

# S2 File. Ovid MEDLINE Search Strategy

| 1 exp Beds/  2 mattress$.mp.  3 cushion$.mp.  4 (foam or transfoam).mp.  5 overlay$.mp.  6 (pad or pads).ti,ab.  7 gel.ti,ab.  8 pressure relie$.mp.  9 pressure reduc$.mp.  10 pressure alleviat$.mp.  11 (low pressure adj2 device$).mp.  12 (low pressure adj2 support).mp.  13 (constant adj2 pressure).mp.  14 static air.mp.  15 (alternat$ adj pressure).mp.  16 air suspension$.mp.  17 air bag$.mp.  18 water suspension$.mp.  19 (elevation adj2 device$).mp.  20 (clinifloat or maxifloat or vaperm or therarest or sheepskin or hammock or foot waffle or silicore or pegasus or cairwave).mp.  21 ((turn$ or tilt$) adj (bed$ or frame$)).mp.  22 (kinetic adj (therapy or table$)).mp.  23 net bed$.mp.  24 (positioning or repositioning).mp.  25 or/1-24  26 exp Pressure Ulcer/  27 (pressure adj (ulcer$ or sore$)).mp.  28 (decubitus adj (ulcer$ or sore$)).mp.  29 (bed adj (ulcer$ or sore$)).mp.  30 or/26-29  31 25 and 30 |
| --- |
