# Supplementary material for: Support surfaces for pressure ulcer prevention: A network meta-analysis
Source: PLoS One. 2018 Feb 23;13(2):e0192707. doi: 10.1371/journal.pone.0192707 (PMC5825032; doi:10.1371/journal.pone.0192707)
Supplement: S3 File — (DOCX) [file pone.0192707.s003.docx]

# S3 File. The detailed steps to define and classify support surfaces

| We followed a classification process as below to define and classify specific support surfaces into meaningful intervention groups.  (1) For each specific support surface in an included study, its “clinically meaningful units” were identified from intervention descriptions presented by the original authors and other accessible information (see Data Extraction section in text). Other characteristics of support surfaces (i.e. manufacturers, costs, and generations) were ignored.  In the review, “clinically meaningful units” refers to components, features, and categories of support surfaces (see below; NPUAP S3I, 2007), which could influence how a support surface operates (Melendez-Torres et al., 2015):   - Categories: i.e. general categories reflecting the pressure redistribution modes of a support surface, the requirement for electrical power for their functionality (i.e. powered or non-powered), and the nature of its presence (i.e. mattress and overlay); - Components: i.e. construction materials, including air-cells, foam, gel, and water etc.; - Features: i.e. functional component of a support surface, including air-fluidised, alternating pressure, low-air-loss, etc.   (2) The support surface was categorised as “reactive” if it worked through redistributing the weight over a maximum body surface area, as “active” if it is designed to inflate and deflate to change the duration of the applied pressure, or as “hybrid” if it includes both “reactive” and “active” modes. After this, component- and feature-related information was added to the “reactive”, “active”, or “hybrid” category in order to specify the support surface in further detail. The requirement re electrical power was also specified.  There is an expectation to the classification process: “standard hospital surfaces” are defined as a group of standard care support surfaces with no reactive (or active) pressure redistribution capabilities or any functional feature (e.g. low-air-loss). |
| --- |

References

Melendez-Torres, G.J., Bonell, C., Thomas, J., 2015. Emergent approaches to the meta-analysis of multiple heterogeneous complex interventions. BMC Med Res Methodol 15, 47.

National Pressure Ulcer Advisory Panel (NPUAP). Support Surface Standards (S3I) Terms and Definitions Related to Support Surfaces. Available at http://www.npuap.org/wp-content/uploads/2012/03/NPUAP_S3I_TD.pdf Access in 15 Dec. 15.
