# Supplementary material for: Support surfaces for pressure ulcer prevention: A network meta-analysis
Source: PLoS One. 2018 Feb 23;13(2):e0192707. doi: 10.1371/journal.pone.0192707 (PMC5825032; doi:10.1371/journal.pone.0192707)
Supplement: S4 File — (DOCX) [file pone.0192707.s004.docx]

# S4 File. Detailed procedures in network meta-analysis (STATA commands in Italic)

1. Understanding evidence base

Use *networkplot* to produce a network plot of intervention groups for understanding the geometry of the evidence base and informing the analysis plan

Use *netweight* to estimate the percentage contribution of each pairwise meta-analysis to the network contrast estimates and to the entire network

1. Performing sensitivity analysis

Perform one sensitivity analysis to assess the impact of missing data imputation (i.e. a complete case analysis for the main analysis, followed by a repeated analysis with missing data added to the denominator but not the numerator), and one sensitivity analysis to assess the impact of removing unpublished studies.

1. Estimating relative effects

Use the *network meta consistency* command to fit a consistency model, which assumed a common heterogeneity across comparisons in the whole network, if global statistical inconsistency was not detected; if detected, an inconsistency model was fitted for the analysis (White et al., 2012).

1. Assessing publication biases

Assess publication biases by inspecting the scope of literature search, retrieving unpublished data, and using the funnel plot (Salanti et al., 2014). We did funnel plot for each pairwise meta-analysis that included more than 10 studies (Peters et al., 2008); and a comparison-adjusted funnel plot for the network to evaluate small-study effects (Chaimani and Salanti 2015). The underlying assumption for the comparison-adjusted funnel plot is that advanced support surfaces are favoured in small studies.

1. Calculating relative rankings

Use the *sucra* command to calculate the relative rankings of intervention groups and present mean ranks, the surface under the cumulative ranking curve (SUCRA) percentages and rankograms (Chaimani and Salanti, 2015).

1. Checking transitivity assumption

Compare descriptive statistics of patient characteristics. Where we failed to detect potential effect modifiers, assume that the transitivity assumption holds true to perform NMA because there is no evidence from the literature suggesting possible intransitivity across comparisons.

1. Assessing heterogeneity and inconsistency

5.1 Use the design-by-treatment interaction model (via *network meta inconsistency*) and the model of Lu and Ades (2006) (via *network meta inconsistency, luades*) to examine **global inconsistency** (White et al., 2012).

5.2 Use node-splitting method for each comparison via *network sidesplit* (Dias et al., 2010) and the Bucher method-based inconsistency plot test for closed loops via *ifplot* (Song et al., 2012) to identify **local inconsistency**. For the *ifplot* test, loop-specific heterogeneity was assumed for its main analysis; and then comparison-specific heterogeneity and common network heterogeneity were assumed for sensitivity analyses (Veroniki et al., 2013).

5.3 Evaluate the common network **heterogeneity** with tau-squared and I^2^ statistic and the 95% CIs of I^2^. Then use *decomp.design* in R to explain whether the common network heterogeneity was attributable to inconsistency or within-study heterogeneity (Krahn et al., 2013). The heterogeneity was considered as low, moderate, or high if I^2^ = 25%, 50%, or 75%, respectively (Higgins et al., 2003).

5.4 When important inconsistency and/or heterogeneity occurred, follow steps proposed by Cipriani et al. (2013) to investigate further: (1) check the data extraction and data entry for errors and possible outlying studies; (2) if outliers existed, perform post hoc sensitivity analysis by removing them; (3) if inconsistency/heterogeneity was still evident, perform post hoc subgroup analyses for four study-level characteristics (see below) in order to **explain heterogeneity/ inconsistency** as much as possible; and (4) if the above methods failed to find possible sources of inconsistency/ heterogeneity, apply the random-effect inconsistency model for network meta-analysis, which allows for additional uncertainty associated with inconsistency/heterogeneity.

5.5 Perform pre-specified subgroup analyses for two potentially known effect modifiers: funding sources (Lexchin et al., 2003) and risk of bias (Schulz et al., 1995), as well as **subgroup analyses** for setting, operating theatre (OR) as setting or not, baseline skin status, and follow-up duration, considering that (1) baseline risk of pressure ulcer development may vary among settings (Bredesen et al., 2015); (2) OR setting: patients undergoing prolonged surgeries commonly require immobilisation with the use of anaesthesia, which may be different from other settings (Pham et al., 2011); (3) baseline skin status was regarded as one of the main risk factors for pressure ulceration (Coleman et al., 2013); and (4) follow-up duration: pressure ulcer incidence seemed to be heterogeneous between different time periods (Kaltenthaler et al., 2001).

References

Bredesen, I.M., Bjøro, K., Gunningberg, L., Hofoss, D., 2015. Patient and organisational variables associated with pressure ulcer prevalence in hospital settings: a multilevel analysis. BMJ Open 5, e007584.

Chaimani, A., Salanti, G., 2015. Visualizing assumptions and results in network meta-analysis: The network graphs package. The Stata Journal 2015; 15 (4): 905–950.

Chaimani, A., Higgins, J.P.T., Mavridis, D., Spyridonos, P., Salanti, G., 2013. Graphical Tools for Network Meta-Analysis in STATA. PLoS One 8. doi:10.1371/journal.pone.0076654

Cipriani, A., Higgins, J.P.T., Geddes, J.R., Salanti, G., 2013. Conceptual and technical challenges in network meta-analysis. Ann. Intern. Med. 159, 130–137.

Coleman, S., Gorecki, C., Nelson, E.A., Closs, S.J., Defloor, T., Halfens, R., Farrin, A., Brown, J., Schoonhoven, L., Nixon, J., 2013. Patient risk factors for pressure ulcer development: systematic review. Int J Nurs Stud 50, 974–1003.

Dias, S., Welton, N.J., Sutton, A.J., Caldwell, D.M., Lu, G., Ades, A.E., 2013. Evidence synthesis for decision making 4: inconsistency in networks of evidence based on randomized controlled trials. Med Decis Making 33, 641–656.

Higgins, J.P.T., Thompson, S.G., Deeks, J.J., Altman, D.G., 2003. Measuring inconsistency in meta-analyses. BMJ 327, 557–560.

Krahn, U., Binder, H., Konig, J., 2013. A graphical tool for locating inconsistency in network meta-analyses. BMC.Med.Res.Methodol. 13, 35. doi:10.1186/1471-2288-13-35

Lu G, Ades AE. Assessing Evidence Inconsistency in Mixed Treatment Comparisons. Journal of the American Statistical Association 2006;101: 447–459.

Peters, J.L., Sutton, A.J., Jones, D.R., Abrams, K.R., Rushton, L., 2008. Contour-enhanced meta-analysis funnel plots help distinguish publication bias from other causes of asymmetry. J Clin Epidemiol 61, 991–996. doi:10.1016/j.jclinepi.2007.11.010

Pham, B., Teague, L., Mahoney, J., Goodman, L., Paulden, M., Poss, J., Li, J., Sikich, N.J., Lourenco, R., Ieraci, L., Carcone, S., Krahn, M., 2011. Support surfaces for intraoperative prevention of pressure ulcers in patients undergoing surgery: a cost-effectiveness analysis. Surgery 150, 122–132.

Salanti, G., Del Giovane, C., Chaimani, A., Caldwell, D.M., Higgins, J.P.T., 2014. Evaluating the Quality of Evidence from a Network Meta-Analysis. PLoS One 9. doi:10.1371/journal.pone.0099682

Song, F., Clark, A., Bachmann, M.O., Maas, J., 2012. Simulation evaluation of statistical properties of methods for indirect and mixed treatment comparisons. BMC Medical Research Methodology 12, 138.

Veroniki, A.A., Vasiliadis, H.S., Higgins, J.P.T., Salanti, G., 2013. Evaluation of inconsistency in networks of interventions. Int J Epidemiol 42, 332–345. doi:10.1093/ije/dys222

White, I.R., Barrett, J.K., Jackson, D., Higgins, J.P.T., 2012. Consistency and inconsistency in network meta-analysis: model estimation using multivariate meta-regression. Res Synth Methods 3, 111–125. doi:10.1002/jrsm.1045
