# Supplementary material for: Support surfaces for pressure ulcer prevention: A network meta-analysis
Source: PLoS One. 2018 Feb 23;13(2):e0192707. doi: 10.1371/journal.pone.0192707 (PMC5825032; doi:10.1371/journal.pone.0192707)
Supplement: S5 File — (DOCX) [file pone.0192707.s005.docx]

# S5 File. Reference list of included studies

1. Andersen 1982

Andersen KE, Jensen O, Kvorning SA, Bach E. Decubitus prophylaxis: a prospective trial on the efficiency of alternating pressure air mattresses and water mattresses. *Acta Dermato-Venereologica (Stockholm)* 1982;63(3):227-30.

1. Aronovitch 1999

Aronovitch SA, Wilber M, Slezak S, Martin T, Utter D. A comparative study of an alternating air mattress for the prevention of pressure ulcers in surgical patients. *Ostomy/Wound Management* 1999;45(3):34-40, 42-4.

1. Bennett 1998

Bennett RG, Baran PJ, DeVone LV, Bacetti H, Kristo B, Tayback M, et al. Low airloss hydrotherapy versus standard care for incontinent hospitalized patients [see comments]. Journal of the American Geriatrics Society 1998;46(5):569-76.

1. Bliss 1967

Bliss MR, McLaren R, Exton-Smith AN. Preventing pressure sores in hospital: controlled trial of a large-celled ripple mattress. Br Med J. 1967 Feb 18;1(5537):394-7.

1. Cao 2013

Juan Cao, YaliTian, Lingli Zhao, et al. Efficacy of ACTION cushion in preventing sacrococcygeal pressure ulcers for elderly ICU patients. Journal of Medical theory and practice. 2013; 26(2): 242-244.

1. Cavicchioli 2007

Cavicchioli A, Carella G. Clinical effectiveness of a low-tech versus high-tech pressure redistributing mattress.*Journal of Wound Care* 2007;16(7):285-9.

1. Chen 2015

Xiaohua Chen, Yong Tao, YulanPeng, et al. Application of three pads in surgeries with prone position. Chinese Journal of Practical Nursing. 2015; 31(14): 1057-1059.

1. Cobb 1997

Cobb GA, Yoder LH, Warren JB. Pressure ulcers: patient outcomes on a KinAir bed or EHOB waffle mattress.TriService Nursing Research Program (TSNRP). Bethesda, Maryland, USA, 1997.

1. Collier 1996

Collier ME. Pressure-reducing mattresses. *Journal of Wound Care* 1996;5(5):207-11.

1. Conine 1990

Conine TA, Daechsel D, Choi AK, Lau MS. Costs and acceptability of two special overlays for the prevention of pressure sores. *Rehabilitation Nursing* 1990;15(3):133-7.

Conine TA, Daechsel D, Lau MS. The role of alternating air and silicore overlays in preventing decubitus ulcers.International Journal of Rehabilitation Research 1990;13(1):57-65.

1. Cooper 1998

Cooper PJ, Gray DG, Mollison J. A randomised controlled trial of two pressure reducing surfaces. *Journal of Wound Care* 1998;7(8):374-6.

1. Daechsel 1985

Daechsel D, Conine TA. Special mattresses: effectiveness in preventing decubitus ulcers in chronic neurologic patients. *Archives of Physical Medicine and Rehabilitation* 1985;66(4):246-8.

1. Demarre 2012

Demarré L, Beeckman D, Vanderwee K, Defloor T, Grypdonck M, Verhaeghe S. Multi-stage versus single-stage inflation and deflation cycle for alternating low pressure air mattresses to prevent pressure ulcers in hospitalised patients: A randomised-controlled clinical trial. *International Journal of Nursing Studies* 2012;49(4):416-426.

1. Economides 1995

Economides NG, Skoutakis VA, Carter CA, Smith VH. Evaluation of the effectiveness of two support surfaces following myocutaneous flap surgery. *Advances in Wound Care* 1995;8(1):49-53.

1. Ewing 1964

Ewing MR, Garrow C, Presley TA, Ashley C, Kinsella NM. Further experiences in the use of sheep skins as an aid in nursing. *The Australian Nurses' Journal* 1964;Sept:215-9.

1. Feuchtinger 2006

Feuchtinger J. Preventing decubitus ulcer in heart surgery interventions: visco-elastic foam layer on the operating room table — a study [Viskoelastische schaumstoffauflage auf dem operationstisch — eine studie]. *Pflege Zeitschrift* 2006;59(8):498-501.

Feuchtinger J, de Bie R, Dassen T, Halfens R. A 4-cm thermoactive viscoelastic foam pad on the operating room table to prevent pressure ulcer during cardiac surgery. Journal of Clinical Nursing 2006;15(2):162-7.

1. Finnegan 2008

Finnegan MJ., Gazzerro L., Finnegan JO., Lo P. Comparing the effectiveness of a specialized alternating air pressure mattress replacement system and an air-fluidized integrated bed in the management of post-operative flap patients: a randomized controlled pilot study. Journal of tissue viability Feb 2008;17(1):2-9.

1. Gao 2014

Miaoli Gao, Hong Xiao. Study of air mattress in reducing local skin pressure status for patients with persistent vegetative state. Int J Nurs 2014; 33(9): 2568-2570.

1. Gray 1994

Gray D. A randomised clinical trial of two foam mattresses. Aberdeen Royal Hospitals NHS Trust.

Gray D. A randomised controlled trial of two foam mattresses. Journal of Tissue Viability 1994;4(3):92.

Gray DG, Campbell M. A randomized clinical trial of two types of foam mattresses. Journal of Tissue Viability1994;4(4):128-32.

Gray DG, Cooper PJ, Campbell M. A study of the performance of a pressure reducing foam mattress after three years of use. Journal of Tissue Viability 1998;8(3):9-13.

1. Gray 1998

Gray D, Smith M. A randomized controlled trial of two pressure-reducing foam mattresses. European Wound Management Association Conference; 1998 November; Harrogate, UK. 1998:4.

Gray DG, Smith M. Comparison of a new foam mattress with the standard hospital mattress. J Wound Care 2000;9(Pt 1):29–31.

1. Gray 2008

Gray D, Cooper P, Bertram M, Duguid K, Pirie G. A clinical audit of the Softform Premier Active[trademark] mattress in two acute care of the elderly wards. Wounds UK 2008;4(4):124-8.

1. Gunningberg 2000

Gunningberg L, Lindholm C, Carlsson M, Sjoden P-O. Effect of visco-elastic foam mattresses on the development of pressure ulcers in patients with hip fractures. Journal of Wound Care 2000;9(10):455-60.

1. Hampton 1997

Hampton S. Evaluation of the new Cairwave Therapy System in one hospital trust. British Journal of Nursing1997;6(3):167-70.

1. Hofman 1994

Hofman A, Geelkerken RH, Wille J, Hamming JJ, Hermans J, Breslau PJ. Pressure sores and pressure-decreasing mattresses: controlled clinical trial. Lancet 1994;343(8897):568-71.

1. Inman 1993

Inman KJ, Sibbald WJ, Rutledge FS, Clark BJ. Clinical utility and cost-effectiveness of an air suspension bed in the prevention of pressure ulcers. JAMA 1993;269(9):1139-43.

1. Ji 2011

Chunping Ji, Jinxia Dong, Chun Li. Comparison of effectiveness of preventing pressure sore by three different mattresses. Chinese Nursing Research 2011; 25(7A): 1728.

1. Jiang 2015

Qixia Jiang, Yajun Zhu, Jing Jia, et al. Randomised controlled trial of comparison of effect of two reduced pressure mattress in preventing pressure ulcer in surgical patients. Nurs J Chin PLA. 2015; 32(5): 20-24, 57.

1. Jolley 2004

Jolley DJ, Wright R, McGowan S, Hickey MB, Campbell DA, Sinclair RD, et al. Preventing pressure ulcers with the Australian Medical Sheepskin: an open-label randomised controlled trial. Medical Journal of Australia2004;180(7):324-7.

1. Kemp 1993

Kemp MG, Kopanke D, Tordecilla L, Fogg L, Shott S, Matthiesen V, et al. The role of support surfaces and patient attributes in preventing pressure ulcers in elderly patients. Research in Nursing and Health 1993;16(2):89-96.

1. Laurent 1998

Laurent S. Effectiveness of pressure decreasing mattresses in cardiovascular surgery patients: a controlled clinical trial. 3rd European Conference for Nurse Managers, 1997 Oct; Brussels, Belgium. Brussels, 1998.

1. Lazzara 1991

Lazzara DJ, Buschmann MBT. Prevention of pressure ulcers in elderly nursing home residents: are special support surfaces the answer?. *Decubitus* 1991;4(4):42-6.

1. Liu 2012

Haiying Liu, Chunyin Su, Xueping Hu, et al. Comparison of decompression effect of the static and dynamic air cushion on bedridden in-patients. Chin J Mod Nurs 2012; 18(36): 4363-4365.

1. Malbrain 2010

Malbrain M., Hendriks B., Wijnands P., Denie D., Jans A., Vanpellicom J., De Keulenaer B. A pilot randomised controlled trial comparing reactive air and active alternating pressure mattresses in the prevention and treatment of pressure ulcers among medical ICU patients. Journal of tissue viability Feb 2010;19(1):7-15

1. McGowan 2000

McGowan S, Montgomery K, Jolley D, Wright R. [The role of sheepskins in preventing pressure ulcers in elderly orthopaedic patients]. First World Wound Healing Congress; 2000, 10-13 September; Melbourne, Australia.2000:108.

McGowan S, Montgomery K, Jolley D, Wright R. The role of sheepskins in preventing pressure ulcers in elderly orthopaedic patients. Primary Intention 2000;8(4):1-8.

1. Mistiaen 2009

Mistiaen P, Francke A, Achterberg W, Ament A, Halfens R, Huizinga J. Australian Medical Sheepskin is effective for the prevention of pressure ulcers [Australische Medische Schapenvacht effectief bij de preventie van stuitdecubitus]. *Tijdschrift voor Ouderengeneeskunde* 2009;5:186-90.

1. Nixon 1998

Bridel-Nixon J, McElveney D, Brown J, Mason S. [Findings from a double-triangular sequential-design randomized clinical trial of a dry polymer pad]. European Wound Management Association Conference; 1997, 27-29 April; Milan, Italy. London: Macmillan Magazines, 1997:20-1.

Bridel-Nixon J, McElveney D, Brown J, Mason S. A randomized controlled trial using a double-triangular sequential design: methodology and management issues. European Wound Management Association Conference; 1997, 27-29 April; Milan, Italy. London: Macmillan Magazines, 1997:65-6.

Nixon J, McElvenny D, Mason S, Brown J, Bond S. A sequential randomised controlled trial comparing a dry visco-elastic polymer pad and standard operating table mattress in the prevention of postoperative pressure sores.International Journal of Nursing Studies 1998;35(4):193-203.

1. Nixon 2006

Iglesias C, Nixon J, Cranny G, Nelson EA, Hawkins K, Phillips A, et al. Pressure relieving support surfaces (PRESSURE) trial: cost effectiveness analysis. BMJ 2006;332(7555):1416.

Nixon J, Cranny G, Iglesias C, Nelson EA, Hawkins K, Phillips A, et al. Randomised, controlled trial of alternating pressure mattresses compared with alternating pressure overlays for the prevention of pressure ulcers: PRESSURE (pressure relieving support surfaces) trial. BMJ 2006;332(7555):1413-5.

Nixon J, Nelson EA, Cranny G, Iglesias CP, Hawkins K, Cullum NA, et al. Pressure relieving support surfaces: A randomised evaluation. Health Technology Assessment (Winchester, England)  2006;10(22):iii-101.

1. Ozyurek 2015

Ozyurek P, Yavuz M. Prevention of pressure ulcers in the intensive care unit: a randomized trial of 2 viscoelastic foam support surfaces. Clin Nurse Spec. 2015;29(4):210-7.

1. Price 1999

Price P, Bale S, Newcombe R, Harding K. Challenging the pressure sore paradigm. *Journal of Wound Care*1999;8(4):187-90.

1. Qu 2014

Xiaolong Qu, Qixia Jiang. Comparison of effectiveness of static air mattress and dynamic air mattress support in preventing pressure ulcers in patients of department of neurology. Nurs J Chin PLA 2014; 31(24): 10-13.

1. Rafter 2011

Rafter L. Evaluation of patient outcomes: pressure ulcer prevention mattresses. British Journal of Nursing 2011;20(11):32.

1. Ricci 2013

Ricci E, Roberto C, Ippolito A, Bianco A, Scalise MT. A new pressure-relieving mattress overlay. *European Wound Management Association Journal* 2013;13(1):27-32.

1. Russell 2000

Dunlop V. Preliminary results of a randomised controlled study of a pressure ulcer prevention system. *Advances in Wound Care* 1998;11(3 suppl 1):14.

Lichtenstein S. A 7 day comparative randomized parallel single centre study to determine the safety and efficacy of the Micropulse system for the prevention of pressure ulcers. Micropulse 1997.

Russell JA, Lichtenstein SL. Randomised controlled trial to determine the safety and efficacy of a multi-cell pulsating dynamic mattress system in the prevention of pressure ulcers in patients undergoing cardiovascular surgery. *Ostomy/Wound Management* 2000;46(2):46-51, 54-5.

1. Russell 2003

Russell LJ, Reynolds TM, Park C, Rithalia S, Gonsalkorale M, Birch J, et al. Randomised clinical trial comparing CONFOR-Med and standard hospital mattresses: results of the prevention of pressure ulcers study (PPUS-1).*Advances in Skin and Wound Care* 2003;16(6):317-27.

1. Sanada 2003

Matsui Y, Miyake S, KawasakiT, Konya C, Sugama J, Sanada H. Randomized controlled trial of a two layer type air cell mattress in the prevention of pressure ulcers. *Japanese Journal of Pressure Ulcers* 2001;3(3):331-7.

Sanada H, Sugama J, Matsui Y, Konya C, Kitagawa A, Okuwa M, et al. Randomised controlled trial to evaluate a new double-layer air-cell overlay for elderly patients requiring head elevation. *Journal of Tissue Viability.* 2003;13(3):112-4, 116, 118 passim.

1. Santy 1994

Santy JE, Butler MK, Whyman JD. A comparison study of 6 types of hospital mattress to determine which most effectively reduces the incidence of pressure sores in elderly patients with hip fractures in a District General Hospital. Report to Northern & Yorkshire Regional Health Authority. 1994.

1. Schultz 1999

Schultz A, Bien M, Dumond K, Brown K, Myers A. Etiology and incidence of pressure ulcers in surgical patients.*AORN Journal* 1999;70(3):434, 437-40, 443-9.

Schultz AA. Study results: prediction and prevention of pressure ulcers in surgical patients. Advances in Wound Care1998;11(3 Suppl):11.

1. Sideranko 1992

Sideranko S, Quinn A. Burns K, Froman RD. Effects of position and mattress overlay on sacral and heel pressures in a clinical population. *Research in Nursing and Health* 1992;15(4):245-51.

1. Stapleton 1986

Stapleton M. Preventing pressure sores -- an evaluation of three products... foam, ripple pads, and Spenco pads.*Geriatric Nursing (London, England)* 1986;6(2):23-5.

1. Takala 1996

Takala J, Varmavuo S, Soppi E. Prevention of pressure sores in acute respiratory failure: a randomised controlled trial. *Clinical Intensive Care* 1996;7(5):228-35.

1. Tang 2014

Jing Tang, Xiuyun Li. Physiotherapy inflatable mattress with replacement sheets on the clinical application of prevention pressure in patients. China Medical Equipment 2014; 11(6): 99-101.

1. Taylor 1999

Taylor L. Evaluating the Pegasus Trinova: a data hierarchy approach. *British Journal of Nursing* 1999;8(12):771-8.

1. Theaker 2005

Theaker C, Kuper M, Soni N. Pressure ulcer prevention in intensive care - a randomised control trial of two pressure-relieving devices. *Anaesthesia* 2005;60(4):395-9.

1. Vanderwee 2005

Vanderwee K, Grypdonck MH, Defloor T. Effectiveness of an alternating pressure air mattress for the prevention of pressure ulcers. *Age and Ageing* 2005;34(3):261-7.

1. van Leen 2011

van Leen M, Hovius S, Neyens J, Halfens R, Schols J. Pressure relief, cold foam or static air? A single center, prospective, controlled randomized clinical trial in a dutch nursing home. *Journal of TIssue Viability* 2011;20:30-34.

1. van Leen 2013

van Leen M, Hovius S, Halfens R, Neyens J, Schols J. Pressure relief with visco-elastic foam or with combined static air overlay? A prospective, crossover randomized clinical trial in a dutch nursing home. Wounds: a compendium of clinical research and practice 2013;25(10):287-292.

1. Vermette 2012

Vermette S, Reeves I, Lemaire J. Cost effectiveness of an air-inflated static overlay for pressure ulcer prevention: A randomized controlled trial. *WOUNDS* 2012;24(8):207.

1. Vyhlidal 1997

Vyhlidal SK, Moxness D, Bosak KS, Van Meter FG, Bergstrom N. Mattress replacement or foam overlay? A prospective study on the incidence of pressure ulcers. *Applied Nursing Research* 1997;10(3):111-20.

1. Wang 2016

Xiaohui Wang, Jiao Jiang, Yuanyuan Wei, et al. Comparing the effect of two types of alternating pressure air mattresses on hospital-acquired pressure ulcers among ICU patients in department of cardiac surgery. Journal of Nursing Science. 2016; 31(4): 12-14.

1. Wei 2016

Jinjin Wei, Zhiyun Guan, Jiyun Hong, et al. Observation on effect of home-made water bag combined with air bed for prevention of pressure ulcer in patients with coma. Chinese Nursing Research. 2016;30(5):1760-1762.

1. Whitney 1984

Whitney JD, Fellows BJ, Larson E. Do mattresses make a difference?. *Journal of Gerontological*

1. Xu 2015

Qin Xu, XiaohuaXie, XiaoxiaoQuan, et al. Comparison of the effect of two kinks of anti-pressure ulcer pad applied to the patints with high risk of pressure ulcer. Journal of Qilu Nursing. 2015; 21(13): 14-16.

1. Zhao 2008

Yuan Zhao, Yi Gong. Application of massage air bed in preventing bedsore of critical patients. 2008; 5(3): 70-71.

1. Zhan 2014

Huiju Zhan, Meiying Liu. Application of cold therapy cushion in preventing pressure sores of unstable pelvic fracture. China’s Modern Medicine and Drugs. 2014; 21(6): 164-166.

1. Zhang 2015

Yuhong Zhang, Qixia Jiang. A comparison of two kinds of decompression scheme to prevent pressure ulcer. Journal of Nursing Science. 2015; 30(17): 36-38.
