# Supplementary material for: Support surfaces for pressure ulcer prevention: A network meta-analysis
Source: PLoS One. 2018 Feb 23;13(2):e0192707. doi: 10.1371/journal.pone.0192707 (PMC5825032; doi:10.1371/journal.pone.0192707)
Supplement: S6 File — (DOCX) [file pone.0192707.s006.docx]

# S6 File. Risk of bias summary

| Study (Reference numbers of included studies) | Selection bias (random sequence generation and allocation concealment) | Performance bias (blinding of participants and personnel) | Detection bias (blinding of outcome assessment) | Attrition bias (incomplete outcome data) | Reporting bias (selective reporting) | Other potential bias (baseline imbalance and differential diagnostic activity) | Overall judgement of study limitation |
| --- | --- | --- | --- | --- | --- | --- | --- |
| Andersen 1982 (1) | Unclear: randomisation and allocation concealment not specified clearly | Unclear: blinding not reported | Unclear: blinding not reported | Unclear: the numbers of randomised participants and drop-outs not reported | Low | Low | No serious limitation |
| Aronovitch 1999 (2) | Unclear: randomisation and allocation concealment not specified clearly | Unclear: blinding not reported | Unclear: blinding not reported | High: high proportion of missing data (25/ 105) in standard hospital surfaces | High: incomplete reporting of participant numbers in analysis | Low | Very serious limitation |
| Bennett 1998 (3) | Low: random-number table;  Unclear: allocation concealment not reported | Unclear: blinding not reported | Unclear: blinding not reported | High: high proportion of missing data (16/116) | High: selective reporting of pressure ulcer data | Low: no imbalance;  High: differential diagnostic activity | Very serious limitation |
| Bliss 1967 (4) | Unclear: randomisation and allocation concealment not reported | Unclear: blinding not reported | Unclear: blinding not reported | High: high proportion of missing data (13/83) | Low | Unclear: baseline balance not clearly specified;  Low: no differential diagnostic activity | Serious limitation |
| Cao 2013 (5) | Low: computer-generated randomisation;  Unclear: allocation concealment not reported | Unclear: blinding not reported | Unclear: blinding not reported | Low | High: outcome not pre-specified | Low | Serious limitation |
| Cavicchioli 2007 (6) | Unclear: randomisation and allocation concealment not reported | Unclear: blinding not reported | Low | High: high proportion of missing data (30/170) | Low | Unclear: unclear baseline balance;  Low: no differential diagnostic activity | Serious limitation |
| Chen 2015 (7) | Low: random-number table;  Unclear: allocation concealment not reported | Unclear: blinding not reported | Unclear: blinding not reported | Low | High: selective reporting of outcome data at specific time-point | Low | Serious limitation |
| Cobb 1997 (8) | Unclear: randomisation not reported;  Low: allocation concealment | Unclear: blinding not reported | Unclear: blinding not reported | Low | Low | High: baseline imbalance and differential diagnostic activity | Serious limitation |
| Collier 1996 (9) | Unclear: randomisation and allocation concealment not reported | High: not blinded | Unclear: blinding not reported | Unclear: not reported | High: outcome data reported incompletely | Unclear; unclear baseline balance;  High: differential diagnostic activity | Very serious limitation |
| Conine 1990 (10) | Unclear: randomisation and allocation concealment not reported | Unclear: blinding not reported | Low | High: high proportion of missing data (39/187) | Low | Low: baseline balance;  Unclear: unclear differential diagnostic activity | Serious limitation |
| Cooper 1998 (11) | Low: “conservatively numbered envelopes” and adequate concealment | Unclear: blinding not reported | Unclear: blinding not reported | High: high proportion of missing data (16/100) | Low | Low | Serious limitation |
| Daechsel 1985 (12) | Unclear: randomisation and allocation concealment not reported | Unclear: blinding not reported | Unclear: blinding not reported | Low | Low | Low | No serious limitation |
| Demarre 2012 (13) | Low: computer-generated randomisation and adequate concealment | High: probably no blinding | High: probably no blinding | Low | Low | Low | Very serious limitation |
| Economides 1995 (14) | Low: random-number table and adequate concealment | Unclear: blinding not reported | Unclear: blinding not reported | Low | Low | Low | No serious limitation |
| Ewing 1964 (15) | Unclear: randomisation and allocation concealment not reported | Unclear: blinding not reported | Unclear: blinding not reported | Unclear: not reported | High: outcome data presented incompletely | Unclear: unclear baseline balance;  Low: no differential diagnostic activity | Serious limitation |
| Feuchtinger 2006 (16) | Unclear: randomisation and allocation concealment not reported | Unclear: blinding not reported | Low | Low: ITT analysis | Low | Low | No serious limitation |
| Finnegan 2008 (17) | Low: Web-based randomisation software;  Unclear: concealment not reported | Unclear: blinding not reported | High: no blinding | High: high proportion of missing data (9/40) | Low | Unclear: baseline balance and diagnostic activity not specified clearly | Very serious limitation |
| Gao 2014 (18) | Low: random-number table;  Unclear: concealment not reported | Unclear: blinding not reported | Unclear: blinding not reported | Low | Unclear: not reported | Low: baseline balance;  Unclear: diagnostic activity not specified clearly | No serious limitation |
| Gray 1994 (19) | Unclear: randomisation and allocation concealment not reported | Unclear: blinding not reported | Unclear: blinding not reported | Unclear: attrition not reported | Low | Unclear: unclear baseline balance;  Low: no differential diagnostic activity | No serious limitation |
| Gray 1998 (20) | Unclear: randomisation and allocation concealment not reported | Unclear: blinding not reported | Low | Unclear: attrition not reported | Low | Low | No serious limitation |
| Gray 2008 (21) | Unclear: randomisation and allocation concealment not reported | Unclear: blinding not reported | Unclear: blinding not reported | Unclear: attrition not reported | Unclear: pre-specified outcome not reported | Unclear: not reported | Serious limitation |
| Gunningberg 2000 (22) | Unclear: randomisation and allocation concealment not reported | Unclear: blinding not reported | Unclear: blinding not reported | Unclear: attrition not reported | Low | Low: baseline balance;  Unclear: diagnostic activity not clearly specified | No serious limitation |
| Hampton 1997 (23) | Unclear: randomisation and allocation concealment not reported | Unclear: blinding not reported | Unclear: blinding not reported | Unclear: attrition not reported | Unclear: not reported | Unclear: not reported | Serious limitation |
| Hofman 1994 (24) | Unclear: randomisation and allocation concealment not reported | High: no blinding | High: no blinding | High: high proportion of missing data (8/44) | Low | Low | Very serious limitation |
| Inman 1993 (25) | Unclear: randomisation and allocation concealment not reported | Unclear: blinding not reported | Unclear: blinding not reported | Low | Low | Low | No serious limitation |
| Ji 2011 (26) | Low: random-number table;  Unclear: concealment not reported | Unclear: blinding not reported | Unclear: blinding not reported | Low | Low | Low | No serious limitation |
| Jiang 2015 (27) | Low: random-number table;  Unclear: concealment not reported | Unclear: blinding not reported | Unclear: blinding not reported | Low | Unclear: pre-specified outcome not reported | Low | No serious limitation |
| Jolley 2004 (28) | Unclear: randomisation not reported;  Low: adequate concealment | High: no blinding | High: no blinding | High: high proportion of missing data (98/539) | Low | Unclear: unclear baseline balance;  Low: no differential diagnostic activity | Very serious limitation |
| Kemp 1993 (29) | Low: random-number table;  Unclear: concealment not reported | Unclear: blinding not reported | Unclear: blinding not reported | Low | Low | Unclear: unclear baseline balance;  Low: no differential diagnostic activity | No serious limitation |
| Laurent 1998 (30) | Unclear: randomisation and allocation concealment not reported | High: no blinding | High: no blinding | Low | Low | Low: baseline balance;  Unclear: diagnostic activity not clearly specified | Very serious limitation |
| Lazzara 1991 (31) | Low: random-number table;  Unclear: concealment not reported | Unclear: blinding not reported | Unclear: blinding not reported | Unclear: attrition not reported | Low | Low | No serious limitation |
| Liu 2012 (32) | Low: random-number table;  Unclear: concealment not reported | Unclear: blinding not reported | Unclear: blinding not reported | Low | High: pre-specified outcome reported incompletely | Low: baseline balance;  Unclear: diagnostic activity not clearly specified | Serious limitation |
| Malbrain 2010 (33) | Low: drawing;  Unclear: concealment not reported | Unclear: blinding not reported | Unclear: blinding not reported | Low | Low | High: baseline imbalance;  Low: no differential diagnostic activity | Serious limitation |
| McGowan 2000 (34) | Unclear: randomisation and allocation concealment not reported | High: no blinding | High: no blinding | Low | Low | Low | Very serious limitation |
| Mistiaen 2009 (35) | Low: computer-generated randomisation and adequate concealment | High: no blinding | High: no blinding | Low | High; outcome data reported incompletely | Low | Very serious limitation |
| Nixon 1998 (36) | Low: stratified randomisation; central allocation | Unclear: blinding not reported | Low | Low | Low | High: baseline imbalance;  Low: no differential diagnostic activity | Serious limitation |
| Nixon 2006 (37) | Low: computer-generated randomisation and adequate concealment | High: no blinding | High: no blinding | Low | Low | Low | Very serious limitation |
| Ozyurek 2015 (38) | Low: computer-generated randomisation and central allocation | High: no blinding | High: no blinding | Unclear: drop-outs not clearly specified | Low | High: baseline imbalance;  Low: no differential diagnostic activity | Very serious limitation |
| Price 1999 (39) | Low: computer-generated randomisation;  Unclear: concealment not reported | Unclear: blinding not reported | High: no blinding | High: high proportion of missing data (30/80) | Low | Low | Very serious limitation |
| Qu 2014 (40) | Low: random-number table;  Unclear: concealment not reported | Unclear: blinding not reported | Unclear: blinding not reported | Low | Low | Low | No serious limitation |
| Rafter 2011 (41) | Unclear: randomisation and concealment not reported | Unclear: blinding not reported | Unclear: blinding not reported | Unclear: the number of randomised participants not reported | Low | Unclear: details not clearly specified | No serious limitation |
| Ricci 2013 (42) | Low: computer-generated randomisation;  Unclear: concealment not reported | Unclear: blinding not reported | Unclear: blinding not reported | Low | Unclear: outcome not clearly specified | Low | No serious limitation |
| Russell 2000 (43) | Unclear: randomisation and concealment not reported | Unclear: blinding not reported | High: no blinding | Low | Low | Low | Serious limitation |
| Russell 2003 (44) | Low: simple randomisation;  Unclear: concealment not reported | High: no blinding | High: no blinding | Low | Low | Low | Very serious limitation |
| Sanada 2003 (45) | Unclear: randomisation and concealment not reported | Unclear: blinding not reported | Unclear: blinding not reported | High: high proportion of missing data (26/108) | High: difference in sample size between Japanese and English copies | Low | Very serious limitation |
| Santy 1994 (46) | Low: random-number table;  Unclear: concealment not reported | Unclear: blinding not reported | Unclear: blinding not reported | Unclear: Unclear number of randomised patients | High: data on specific support surface not reported | Low | Serious limitation |
| Schultz 1999 (47) | Low: random-number table;  High: no concealment | Unclear: blinding not reported | Low | Low | Low | Low | Serious limitation |
| Sideranko 1992 (48) | Unclear: randomisation and concealment not reported | Unclear: blinding not reported | Unclear: blinding not reported | Unclear: the number of participants in analysis not specified | Low | Low: baseline balance;  Unclear: diagnostic activity not specified clearly | No serious limitation |
| Stapleton 1986 (49) | Unclear: randomisation and concealment not reported | Unclear: blinding not reported | Unclear: blinding not reported | Low | Unclear: outcome not clearly specified | Low: baseline balance;  Unclear: diagnostic activity not specified clearly | No serious limitation |
| Takala 1996 (50) | Unclear: randomisation not reported;  High: no concealment | High: no blinding | Unclear: blinding not reported | Low | Low | Low | Very serious limitation |
| Tang 2014 (51) | Low: random-number table;  Unclear: concealment not reported | Unclear: blinding not reported | Unclear: blinding not reported | Low | Low | Low: baseline balance;  Unclear: diagnostic activity not specified clearly | No serious limitation |
| Taylor 1999 (52) | Unclear: randomisation not reported;  Low: adequate concealment | Unclear: blinding not reported | Unclear: blinding not reported | Unclear: drop-outs not clearly specified | High: outcome reported only for one group | Low | Serious limitation |
| Theaker 2005 (53) | Unclear: randomisation not reported;  Low: adequate concealment | High: no blinding | High: no blinding | Low | Low | Low | Very serious limitation |
| Vanderwee 2005 (54) | Low: computer-generated randomisation and adequate concealment | Unclear: blinding not reported | Unclear: blinding not reported | Low | Unclear: assessment not specifically for pre-specified outcome | Low | No serious limitation |
| van Leen 2011 (55) | Unclear: randomisation and concealment not reported | Unclear: blinding not reported | Low: no blinding | Low | Low | High: baseline imbalance;  Low: no differential diagnostic activity | Serious limitation |
| van Leen 2013 (56) | Unclear: randomisation and concealment not reported | Unclear: blinding not reported | Unclear: blinding not reported | Low | Low | Low | No serious limitation |
| Vermette 2012 (57) | Unclear: randomisation not reported;  High: no concealment | High: no blinding | High: no blinding | Low | Low | High: baseline imbalance;  Low: no differential diagnostic activity | Very serious limitation |
| Vyhlidal 1997 (58) | Low: drawing;  Unclear: concealment not reported | Unclear: blinding not reported | Unclear: blinding not reported | Low | Low | High: baseline imbalance;  Unclear: diagnostic activity not specified clearly | Serious limitation |
| Wang 2016 (59) | Low: random-number table;  Unclear: concealment not reported | Unclear: blinding not reported | Unclear: blinding not reported | Low | Low | Low: no imbalance;  Unclear: diagnostic activity not clearly specified | No serious limitation |
| Wei 2016 (60) | Low: random-number table;  Unclear: concealment not reported | Unclear: blinding not reported | Unclear: blinding not reported | Low | Low | Low | No serious limitation |
| Whitney 1984 (61) | Unclear: randomisation not reported;  Low: adequate concealment | Unclear: blinding not reported | Unclear: blinding not reported | Low | Low | Unclear: unclear baseline imbalance;  Low: no differential diagnostic activity | No serious limitation |
| Xu 2015 (62) | Low: coin tossing;  Unclear: concealment not reported | Unclear: blinding not reported | Unclear: blinding not reported | Low | Low | Low: no imbalance;  Unclear: diagnostic activity not clearly specified | No serious limitation |
| Zhao 2008 (63) | Low; coin tossing;  Unclear: concealment not reported | Unclear: blinding not reported | Unclear: blinding not reported | Low | Low | Low | No serious limitation |
| Zhan 2014 (64) | Low; random-number table;  Unclear: concealment not reported | Unclear: blinding not reported | Unclear: blinding not reported | Low | Low | Low: no imbalance;  Unclear: diagnostic activity not specified clearly | No serious limitation |
| Zhang 2015 (65) | Low; random-number table;  Unclear: concealment not reported | Unclear: blinding not reported | Unclear: blinding not reported | Low | Low | Low | No serious limitation |
