# Supplementary material for: Support surfaces for pressure ulcer prevention: A network meta-analysis
Source: PLoS One. 2018 Feb 23;13(2):e0192707. doi: 10.1371/journal.pone.0192707 (PMC5825032; doi:10.1371/journal.pone.0192707)
Supplement: S8 File — (DOCX) [file pone.0192707.s008.docx]

**S8 File. Sensitivity analysis of missing data and unpublished data imputation^*^**

| pnpReLAL | 0.66 (0.29, 1.49) (1 study; 123 participants) |  |  |  |  |  |  |  |  |  |  |  | 0.66 (0.08, 5.30) (2 studies; 216 participants) |
| --- | --- | --- | --- | --- | --- | --- | --- | --- | --- | --- | --- | --- | --- |
| 1.29 (0.56,2.94) | pnpReAir | 1.00 (0.20, 4.95) (1 study; 12 participants) |  |  | 1.09 (0.34, 3.56) (1 study; 1074 participants) |  | 0.68 (0.30, 1.54) (5 studies; 506 participants) | 0.43 (0.04, 4.29) (1 study; 37 participants) |  | 1.25 (0.56, 2.77) (1 study; 66 participants) | 0.32 (0.09, 1.13) (2 studies; 146 participants) |  | 0.20 (0.05, 0.75) (2 studies; 123 participants) |
| 1.29 (0.18,9.36) | 1.00 (0.16,6.08) | pReAirfluid |  |  |  |  |  |  |  |  |  |  |  |
| 3.74 (0.51,27.52) | 2.91 (0.42,20.07) | 2.91 (0.21,40.91) | pHybridLAL | 0.53 (0.15, 1.94) (1 study; 62 participants) |  |  |  |  |  |  |  |  |  |
| 1.99 (0.56,7.11) | 1.55 (0.48,4.98) | 1.55 (0.18,13.31) | 0.53 (0.11,2.49) | pHybridAir |  |  | 0.46 (0.20, 1.09) (3 studies; 304 participants) |  |  |  |  |  |  |
| 1.22 (0.25,5.82) | 0.95 (0.25,3.60) | 0.95 (0.10,8.93) | 0.33 (0.03,3.40) | 0.61 (0.10,3.58) | pActLAL |  |  |  |  | 3.00 (0.12, 72.53) (1 study; 156 participants) |  |  |  |
| 0.66 (0.20,2.21) | 0.51 (0.17,1.58) | 0.51 (0.06,4.29) | 0.18 (0.02,1.39) | 0.33 (0.08,1.31) | 0.54 (0.09,3.09) | pActAir npReFoam | 1.04 (0.46, 2.36) (1 study; 157 participants) |  |  |  | 0.89 (0.40, 1.96) (1 study; 152 participants) |  | 0.74 (0.35, 1.57) (1 study; 157 participants) |
| 0.93 (0.41,2.09) | 0.72 (0.38,1.37) | 0.72 (0.11,4.90) | 0.25 (0.04,1.54) | 0.47 (0.18,1.24) | 0.76 (0.17,3.34) | 1.41 (0.53,3.75) | pActAir | 1.21 (0.52, 2.83) (2 studies; 358 participants) |  |  | 0.91 (0.67, 1.25) (4 studies; 719 participants) | 0.90 (0.68, 1.19) (3 studies; 285 participants) | 0.33 (0.23, 0.48) (9 studies; 1309 participants) |
| 1.14 (0.35,3.73) | 0.89 (0.30,2.63) | 0.89 (0.11,7.30) | 0.31 (0.04,2.37) | 0.57 (0.15,2.21) | 0.94 (0.17,5.22) | 1.74 (0.46,6.50) | 1.23 (0.48,3.13) | npReWater |  |  |  |  | 0.35 (0.15, 0.79) (1 study; 316 participants) |
| 0.70 (0.28,1.79) | 0.55 (0.23,1.28) | 0.55 (0.07,4.02) | 0.19 (0.03,1.31) | 0.35 (0.11,1.16) | 0.58 (0.12,2.80) | 1.07 (0.35,3.25) | 0.76 (0.39,1.49) | 0.62 (0.21,1.83) | npReSheepskin |  |  |  | 0.54 (0.30, 0.96) (3 studies; 1424 participants) |
| 1.72 (0.46,6.39) | 1.34 (0.47,3.79) | 1.34 (0.17,10.76) | 0.46 (0.05,4.07) | 0.86 (0.19,4.03) | 1.42 (0.29,6.92) | 2.62 (0.58,11.81) | 1.86 (0.57,6.11) | 1.51 (0.34,6.61) | 2.45 (0.66,9.08) | npReGel | 0.40 (0.02, 8.12) (1 study; 168 participants) |  |  |
| 0.58 (0.26,1.29) | 0.45 (0.23,0.87) | 0.45 (0.07,3.09) | 0.16 (0.02,1.00) | 0.29 (0.10,0.84) | 0.48 (0.11,2.10) | 0.88 (0.34,2.32) | 0.63 (0.42,0.93) | 0.51 (0.19,1.33) | 0.82 (0.44,1.56) | 0.34 (0.10,1.11) | npReFoam | 1.17 (0.64, 2.14) (1 study; 68 participants) | 0.74 (0.49, 1.13) (8 studies; 3009 participants) |
| 0.81 (0.30,2.18) | 0.63 (0.27,1.49) | 0.63 (0.09,4.66) | 0.22 (0.03,1.48) | 0.41 (0.13,1.28) | 0.67 (0.14,3.25) | 1.24 (0.40,3.81) | 0.88 (0.49,1.58) | 0.71 (0.24,2.13) | 1.16 (0.48,2.76) | 0.47 (0.13,1.76) | 1.40 (0.72,2.72) | npReFibre |  |
| 0.38 (0.18,0.80) | 0.29 (0.15,0.56) | 0.29 (0.04,2.00) | 0.10 (0.02,0.65) | 0.19 (0.07,0.54) | 0.31 (0.07,1.36) | 0.58 (0.22,1.51) | 0.41 (0.28,0.60) | 0.33 (0.13,0.85) | 0.54 (0.31,0.93) | 0.22 (0.07,0.72) | 0.65 (0.47,0.90) | 0.47 (0.24,0.91) | SC |

* The table shares the same legend with Table 4 in the text.
