# Supplementary material for: Support surfaces for pressure ulcer prevention: A network meta-analysis
Source: PLoS One. 2018 Feb 23;13(2):e0192707. doi: 10.1371/journal.pone.0192707 (PMC5825032; doi:10.1371/journal.pone.0192707)
Supplement: S9 File — (DOCX) [file pone.0192707.s009.docx]

# S9 File. Quality of evidence assessment

## Summary of findings table for the prevention network

| Comparisons of intervention groups with standard hospital support surfaces | **Anticipated absolute effects^*^** (95% CI) | | Relative effect (95% CI) | Quality of the evidence (GRADE) |
| --- | --- | --- | --- | --- |
|  | **Risk with standard hospital support surfaces** | **Risk with support surfaces** |  |  |
| Non-powered reactive fibre surfaces (npReFibre) | 219 per 1,000 | **103 per 1,000** (53 to 206) | **RR 0.47** (0.24 to 0.94) | ⨁⨁◯◯ LOW ^a,b^ |
| Non-powered reactive foam surfaces (npReFoam) | 219 per 1,000 | **145 per 1,000** (105 to 204) | **RR 0.66** (0.48 to 0.93) | ⨁◯◯◯ VERY LOW ^b,c,d^ |
| Non-powered reactive gel surfaces (npReGel) | 219 per 1,000 | **57 per 1,000** (18 to 193) | **RR 0.26** (0.08 to 0.88) | ⨁◯◯◯ VERY LOW ^a,b,c^ |
| Non-powered reactive sheepskin surfaces (npReSheepskin) | 219 per 1,000 | **118 per 1,000** (68 to 210) | **RR 0.54** (0.31 to 0.96) | ⨁◯◯◯ VERY LOW ^b,e,f^ |
| Non-powered reactive water surfaces (npReWater) | 219 per 1,000 | **74 per 1,000** (28 to 193) | **RR 0.34** (0.13 to 0.88) | ⨁⨁◯◯ LOW ^b,g^ |
| Powered active air surfaces (pActAir) | 219 per 1,000 | **92 per 1,000** (64 to 138) | **RR 0.42** (0.29 to 0.63) | ⨁⨁⨁◯ MODERATE ^h^ |
| Powered active air surfaces plus non-powered reactive foam surfaces (pActAirnpReFoam) | 219 per 1,000 | **129 per 1,000** (48 to 342) | **RR 0.59** (0.22 to 1.56) | ⨁◯◯◯ VERY LOW ^c,g,i^ |
| Powered active low-air-loss surfaces (pActLAL) | 219 per 1,000 | **72 per 1,000** (18 to 326) | **RR 0.33** (0.08 to 1.49) | ⨁◯◯◯ VERY LOW ^a,c,i^ |
| Powered hybrid air surfaces (pHybridAir) | 219 per 1,000 | **48 per 1,000** (15 to 145) | **RR 0.22** (0.07 to 0.66) | ⨁⨁⨁◯ MODERATE ^a^ |
| Powered hybrid low-air-loss surfaces (pHybridLAL) | 219 per 1,000 | **26 per 1,000** (4 to 171) | **RR 0.12** (0.02 to 0.78) | ⨁⨁◯◯ LOW ^a,b^ |
| Powered reactive airfluidised surfaces (pReAirfluid) | 219 per 1,000 | **68 per 1,000** (9 to 466) | **RR 0.31** (0.04 to 2.13) | ⨁◯◯◯ VERY LOW ^a,c,i^ |
| Powered/non-powered reactive air surfaces (pnpReAir) | 219 per 1,000 | **68 per 1,000** (35 to 129) | **RR 0.31** (0.16 to 0.59) | ⨁⨁◯◯ LOW ^c,g^ |
| Powered/non-powered reactive low-air-loss surfaces (pnpReLAL) | 219 per 1,000 | **92 per 1,000** (44 to 197) | **RR 0.42** (0.20 to 0.90) | ⨁◯◯◯ VERY LOW ^b,c,f^ |
| * **Risk with standard hospital support surfaces** was the median risk across studies that provided data for the outcome. **The risk in the intervention group** (and its 95% confidence interval) is based on the assumed risk in the comparison group and the **relative effect** of the intervention (and its 95% CI).   **CI:** Confidence interval; **RR:** Risk ratio | | | | |
| **GRADE Working Group grades of evidence** **High quality:** We are very confident that the true effect lies close to that of the estimate of the effect **Moderate quality:** We are moderately confident in the effect estimate: The true effect is likely to be close to the estimate of the effect, but there is a possibility that it is substantially different **Low quality:** Our confidence in the effect estimate is limited: The true effect may be substantially different from the estimate of the effect **Very low quality:** We have very little confidence in the effect estimate: The true effect is likely to be substantially different from the estimate of effect | | | | |

a. no direct evidence; moderate common network heterogeneity; not covered by an inconsistent loop (downgraded by one level)

b. CI crossed RR value of 0.75 or 1.25 (downgraded by one level)

c. serious limitations (downgraded by one level)

d. high heterogeneity (I-squared > 75%) in pairwise meta-analysis; associated moderate common network heterogeneity, and an inconsistent loop (downgraded by two levels)

e. very serious limitations (downgraded by two levels)

f. high heterogeneity (I-squared > 75%) in pairwise meta-analysis; moderate common heterogeneity in the network; not covered by an inconsistent loop (downgraded by one level)

g. direct evidence; moderate common network heterogeneity; not covered by an inconsistent loop (downgraded by one level)

h. moderate common network heterogeneity; covered by an inconsistent loop; but low heterogeneity in pairwise meta-analysis (downgraded by one level)

i. CI crossed RR values of 0.75 and 1.25 and very wide (downgraded by two levels)

## Confidence in ranking of intervention groups regarding pressure ulcer incidence

**Risk of bias**: the quality was downgraded by one level due to serious study limitations in the entire network;

**Indirectness**: we did not downgrade the quality in indirectness because transitivity assumption was assumed to hold;

**Inconsistency**: the quality for the entire network was downgraded once due to (1) moderate heterogeneity; (2) the possible presence of inconsistent loops; but (3) no global inconsistency in the whole network;

**Imprecision**: the quality was downgrade once due to imprecision in ranking probabilities: only one intervention group had a SUCRA probability over 80% and thus the probabilities for almost all support surfaces to rank low or high are badly differentiated from each other;

**Publication bias**: the quality was not downgraded in publication bias because a comprehensive search was performed and unpublished data were searched even though the comparison-adjusted funnel plot suggested a possibility of publication bias in the whole network (i.e. small studies tend to exaggerate the effects of newer or advanced interventions compared to old or less advanced ones; see Appendix figure 1).

In summary, the quality of evidence for network rankings was very low.


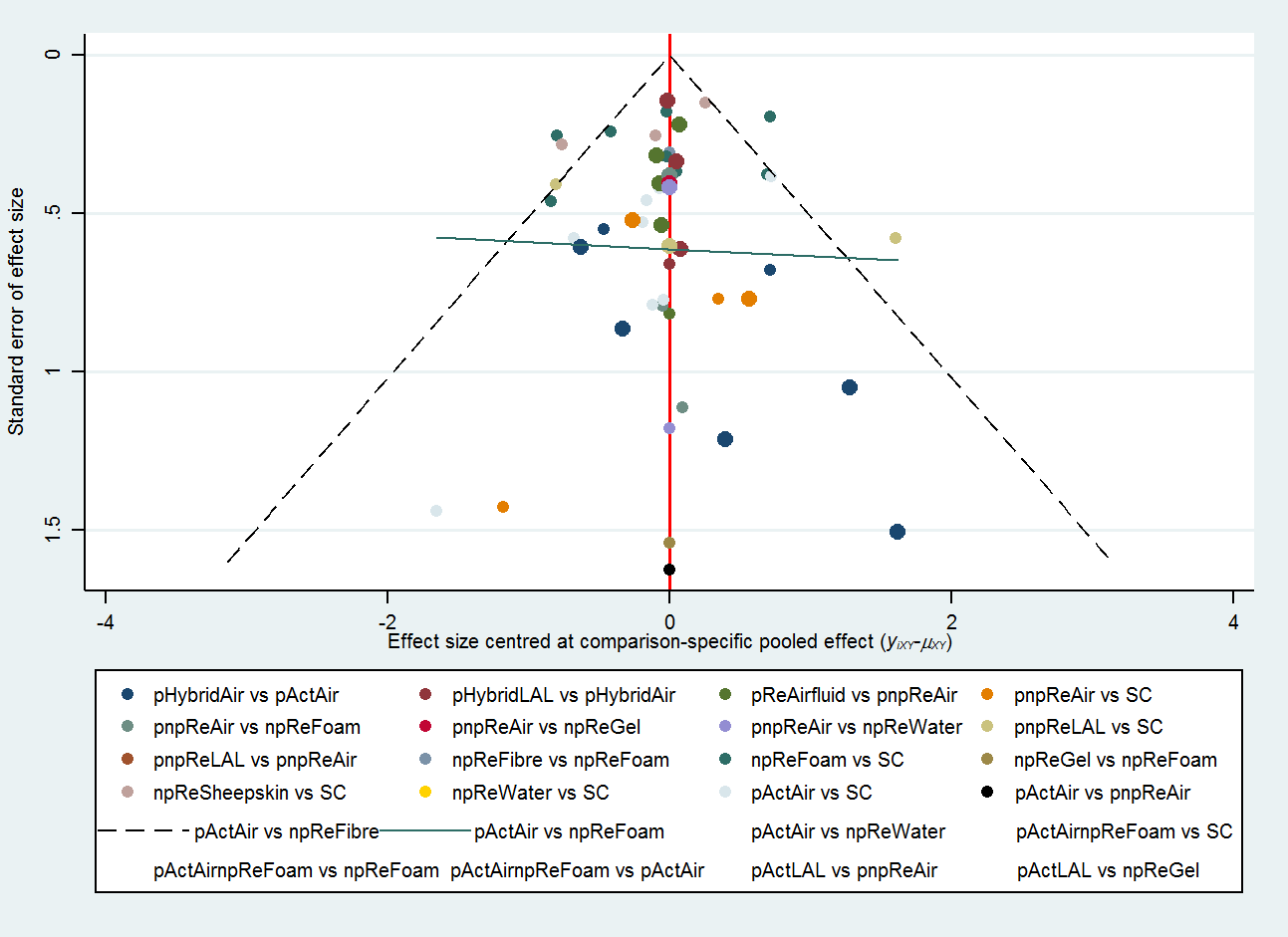


**Appendix figure 1: Comparison-adjusted funnel plot for the prevention network**.
