# Supplementary material for: Support surfaces for pressure ulcer prevention: A network meta-analysis
Source: PLoS One. 2018 Feb 23;13(2):e0192707. doi: 10.1371/journal.pone.0192707 (PMC5825032; doi:10.1371/journal.pone.0192707)
Supplement: S10 File — (DOCX) [file pone.0192707.s010.docx]

# S10 File. Heterogeneity and inconsistency assessment in the prevention network

## Heterogeneity in NMA

The magnitude of the network common heterogeneity, tau-squared (tau2), is 0.195 and the extent of heterogeneity (I^2^) is 56% (95% CI 36 to 70%), which suggest the presence of a moderate heterogeneity.

The test of heterogeneity/inconsistency in R showed that the sum of between-study heterogeneity was high (Q-statistic = 61.09, d.f. = 22, p-value < 0.0001); and there is no inconsistency (Q-statistic = 18.51, d.f. = 13, p-value = 0.139). When exploring between-study heterogeneity in further, three pairwise meta-analyses with significant Q-test results contributed the most to the network heterogeneity: SC vs npReFoam (Q-statistic = 32.76, d.f. = 6, p-value < 0.0001); SC vs npReSheepskin (Q-statistic = 10.13, d.f. = 2, p-value = 0.006); and SC vs pnpReLAL (Q-statistic = 11.60, d.f. = 1, p-value = 0.001).

Overall heterogeneity test suggests that the moderate network heterogeneity results from high between-study heterogeneity in npReFoam vs SC, npReSheepskin vs SC, and pnpReLAL vs SC comparisons. There is no inconsistency in the network.

## Inconsistency in NMA

### Global approach

The global inconsistency test did not suggest the presence of inconsistency in the network (tau-squared = 0.241; Wald test: Chi2(13) = 9.07, p = 0.767).

### Loop-specific approach


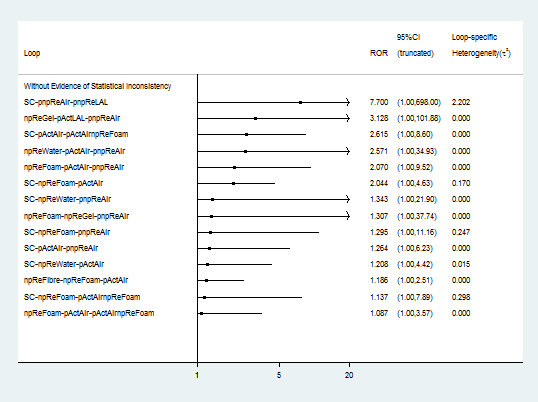


**Appendix figure 2: Inconsistency plot with loop-specific heterogeneity (main analysis).** SC-pnpReAir-pnpReLAL has a ROR of 7.70 and tau-squared of 2.20, suggesting that direct estimate could be seven times as large as the indirect estimate or vice versa (Chaimani et al., 2013).


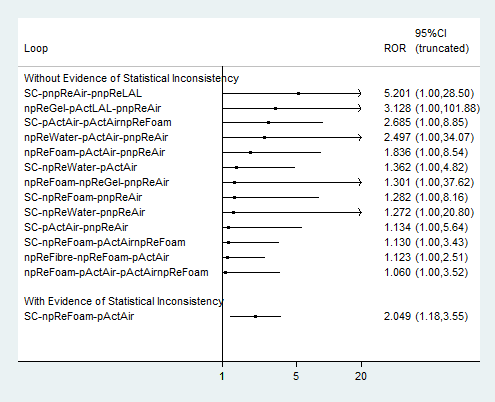


**Appendix figure 3: Inconsistency plot with the common network heterogeneity (tau2 = 0.195) (sensitivity analysis)**. This figure suggests a possible inconsistent loop (i.e. SC-npReFoam-pActAir) of 14 triangular loops, which might be associated with the high heterogeneity of SC vs npReFoam comparison.

References

Chaimani, A., Higgins, J.P.T., Mavridis, D., Spyridonos, P., Salanti, G., 2013. Graphical Tools for Network Meta-Analysis in STATA. PLoS One 8. doi:10.1371/journal.pone.0076654
