# Supplementary material for: Support surfaces for pressure ulcer prevention: A network meta-analysis
Source: PLoS One. 2018 Feb 23;13(2):e0192707. doi: 10.1371/journal.pone.0192707 (PMC5825032; doi:10.1371/journal.pone.0192707)
Supplement: S11 File — (DOCX) [file pone.0192707.s011.docx]

# S11 File. Subgroup analyses

| Analysis | tau | Tau-squared |
| --- | --- | --- |
| Consistency model NMA | 0.441 | 0.195 |
| Subgroup: funding sources (public vs. public & industry vs. industry vs. no/unclear funding) | 0.400 | 0.160 |
| Subgroup: overall risk of bias (very serious vs. serious vs. no serious study limitation) | 0.441 | 0.195 |
| Subgroup: setting (accident and emergency departments and acute care vs. intensive care units vs. general medical wards vs. orthopaedic centres vs. operating theatres vs. long-term care settings) | 0. 498 | 0.248 |
| Subgroup: considering OR as setting or not (yes vs. no) | 0. 400 | 0.160 |
| Subgroup: baseline skin status (unclear vs. no existing pressure ulcers vs. grade 1 ulcers vs. grade 2 or above ulcers vs. existing skin breakdown) | 0. 359 | 0.129 |
| Meta-regression: follow-up in days (continuous data) | 0.422 | 0.178 |
